# Lactylation of PTBP1 drives a pro-apoptotic positive feedback loop in microglia following oxygen-glucose deprivation/reoxygenation-induced injury

**DOI:** 10.1038/s41419-026-08921-9

**Published:** 2026-05-28

**Authors:** Haiyue Zhou, Sutong Xu, Chenming Liu, Qiulu Liu, Yali Wang, Bei Zhang, Chengyu Lv, Yuping Luo, Siguang Li, Chun Li

**Affiliations:** 1 Key Laboratory of Spine and Spinal Cord Injury Repair and Regeneration of Ministry of Education, Tongji Hospital, School of Medicine, Tongji University, Shanghai, China; 2 Stem Cell Research Center, School of Medicine, Tongji University, Shanghai, China

**Keywords:** Microglia, Apoptosis, Acetylation

## Abstract

Cerebral hypoxia-ischemia disrupts cellular energy metabolism and exacerbates microglial apoptosis through mechanisms that remain elusive. In this study, given the substantial lactate accumulation under hypoxic-ischemic conditions, we explored how lactylation, a lactate-derived post-translational modification, drives apoptotic signaling to identify potential therapeutic targets. Global lactylome profiling of microglia subjected to oxygen-glucose deprivation/reoxygenation (OGD/R) revealed widespread protein hyperlactylation, involving 2 555 lactylated sites across 1 071 proteins. Notably, we identified the RNA-binding splicing regulator PTBP1 as a novel non-histone target lactylated at lysine residues K258 and K452 in a manner dynamically regulated by the delactylase Sirt1 and functionally correlated with the induction of microglial apoptotic signaling. Mechanistically, hyperlactylated PTBP1 directly suppressed the expression of USP18, triggering FTO protein degradation and subsequent downregulation of delactylase SIRT1. This loss of SIRT1-mediated lactylate removal further amplified PTBP1 lactylation, ultimately exacerbating apoptotic activation and establishing a self-reinforcing pro-apoptotic positive feedback loop. Consistently, lactylation-deficient mutations (K258R and K452R) on PTBP1 significantly attenuated apoptotic signaling. Our findings delineate a lactylation-driven signaling cascade centered on PTBP1 that critically mediates OGD/R-induced microglial apoptosis, and propose therapeutic targeting of the lactylation-governed PTBP1-USP18-FTO-SIRT1 signaling axis as a promising strategy to ameliorate apoptosis-related neurological pathogenesis.

## Introduction

Cerebral hypoxia and ischemia constitute critical pathological features of ischemic stroke, traumatic brain injury, and related neurological disorders [1–3]. Despite their clinical prevalence, both therapeutic interventions and mechanistic insights remain inadequate. The initial cerebral hypoxic-ischemic injury initiates a multifaceted pathological cascade of secondary injury processes [4], including neuroinflammation [5], apoptosis and programmed necrosis [6–8], excitotoxicity [8, 9], autophagy dysfunction [10], and oxidative stress [11], etc. A comprehensive understanding of these mechanisms is therefore essential, not only to decipher the pathophysiology of cerebral hypoxic-ischemic disorders but also to facilitate the development of neuroprotective strategies.

As the resident immune cells of the central nervous system (CNS) [12, 13], microglia are primary responders to cerebral hypoxia and ischemia, playing a pivotal role in neuroinflammation, oxidative stress, and cell death pathways [12, 14, 15], thereby orchestrating the triggered pathological cascade. This pathological progression is invariably accompanied by significant dysregulation of cellular energy metabolism, predominantly characterized by the aberrant accumulation of lactate resulting from accelerated glycolysis [16, 17]. While lactate serves dual roles as a metabolic intermediate and an immunomodulatory signaling molecule [18, 19], its pathological accumulation exhibits a pronounced link to cellular apoptosis [20, 21]. Emerging evidence indicated that elevated lactate triggered programmed cell death through multipathway mechanisms, encompassing AMPK/Akt/mTOR-mediated apoptosis in dopaminergic neurons [22] and endoplasmic reticulum stress-dependent apoptosis via the ATF4-CHOP axis and caspase-12 activation [23]. Clinically, elevated lactate levels are strongly associated with adverse neurological outcomes in patients following cerebral hypoxic-ischemic injury [24–26], and therapeutic strategies targeting energy metabolic dysfunction have demonstrated efficacy in promoting functional recovery [26, 27]. Nevertheless, the regulatory mechanisms and functional impact of lactate signaling in microglia under cerebral hypoxic-ischemic conditions remain incompletely elucidated.

In this study, leveraging comprehensive lactylome profiling of BV2 microglia under oxygen-glucose deprivation/reoxygenation (OGD/R) conditions, we identified polypyrimidine tract-binding protein 1 (PTBP1) as a novel lactylation target, with site-specific modifications occurring at lysine residues K258 and K452. This lactylation event promoted microglial apoptosis by initiating a USP18-FTO-SIRT1-mediated positive feedback loop. Notably, ablation of PTBP1 lactylation through site-specific mutagenesis (K258R/K452R) significantly attenuated apoptosis and restored cellular viability in OGD/R-treated BV2 microglia, thereby establishing the lactylation-dependent PTBP1 regulatory axis as a critical mechanistic driver of cerebral hypoxic-ischemic injury.

## Materials and methods

### Mice

Male C57BL/6 J mice (8-week-old) were purchased from Shanghai SLAC Laboratory Animal Company and group-housed under specific pathogen-free conditions in a temperature- and humidity-controlled environment with a 12-hour light/dark cycle. All experimental procedures were conducted in accordance with protocols approved by the Animal Ethics Committee of Tongji University.

### BV2 and HMC3 microglial cell cultures

The mouse BV2 and human HMC3 microglial cell lines were cultured in Dulbecco’s Modified Eagle Medium (DMEM) and Minimum Essential Medium (MEM), respectively, each supplemented with 10% heat-inactivated fetal bovine serum (FBS; Gibco) and 1% penicillin/streptomycin (Sigma-Aldrich). Cells were maintained in a thermostatic incubator at 37 °C containing 5% CO₂. Subculturing was performed every 1-2 days upon reaching approximately 80–90% confluence. All cultures routinely tested negative for mycoplasma contamination using PCR-based detection.

### Middle cerebral artery occlusion (MCAO) model

The ischemic stroke model was established through permanent occlusion of the right middle cerebral artery (MCA). Briefly, mice were anesthetized with sodium pentobarbital (50 mg/kg), followed by surgical exposure of the right common carotid artery (CCA), internal carotid artery (ICA), and external carotid artery (ECA). A monofilament nylon suture (1622A4; Beijing Cinontech, China) was then introduced into the ICA and advanced to occlude the origin of the MCA. After 30 minutes of ischemia, the filament was withdrawn to allow reperfusion. Ipsilateral cerebral cortical tissues were collected at 12, 24, and 72 h following reperfusion for further analysis. No randomization method was used to allocate animals to experimental groups. Animals of similar age, sex, and body weight were selected to minimize potential confounding factors. Blinding was not feasible in this study due to the nature of the MCAO model.

### Oxygen-glucose deprivation/reoxygenation (OGD/R) model

The culture medium was removed and BV2 microglial cells were washed with phosphate-buffered saline (PBS; Gibco). To induce oxygen-glucose deprivation (OGD), cells were incubated in glucose-free DMEM and transferred to an anaerobic chamber for 2, 4, 6, or 8 h. Following OGD exposure, the glucose-free medium was removed and replaced with complete DMEM. The cells were then returned to standard culture conditions (37 °C, 5% CO₂) for 24 h to simulate reperfusion. BV2 cells in the control group underwent identical handling without exposure to OGD.

### Transfection of BV2 cells using siRNA and mRNA constructs

Small interfering RNAs (siRNAs) targeting Usp18, Fto, and Sirt1 were obtained from He Yuan Bio. Flag-tagged wild-type PTBP1and its mutant variant (K258R/K452R) mRNAs, along with CALNP™ RNAi and CALNP™ mRNA transfection reagents for in vitro applications, were sourced from D-nano Therapeutics. BV2 cells were seeded in 6-well plates at approximately 20–30% confluence and cultured under standard culture conditions (37 °C, 5% CO₂) for 24 h. Transfection complexes were prepared by incubating each si-RNA or mRNA with the appropriate transfection reagent according to the manufacturer’s instructions, followed by delivery of these complexes to the cells. After 24 h, the transfection medium was replaced with fresh complete medium. The sequences of all siRNA and mRNA oligonucleotides used in this study are provided in Table 1 and Table 2.

**Table 1:** Sequence of the siRNAs.

Name	Species	Forward (5’-3’)	Reverse (5’-3’)
siSirt1-1	mouse	CUUCGAAAUUAUACUCAAATT	UUUGAGUAUAAUUUCGAAGTT
siSirt1-2	mouse	GGCCUAAUAGACUUGCAAATT	UUUGCAAGUCUAUUAGGCCTT
siSirt1-3	mouse	GGGAUCAAGAGGUUGUUAATT	UUAACAACCUCUUGAUCCCTT
siFto-1	mouse	GCACCUACAAGUACUUGAATT	UUCAAGUACUUGUAGGUGCTT
siFto-2	mouse	GGACGAGUCCAGCUUCGAATT	UUCGAAGCUGGACUCGUCCTT
siFto-3	mouse	CGGUGCUCCGUGAAGUUAATT	UUAACUUCACGGAGCACCGTT
siUsp18-1	mouse	GCUCAACUCUACCUUACAATT	UUGUAAGGUAGAGUUGAGCTT
siUsp18-2	mouse	GAAAGUGGUUCUGCUUCAATT	UUGAAGCAGAACCACUUUCTT
siUsp18-3	mouse	CGUCCAGCCCAAAGAGUUATT	UAACUCUUUGGGCUGGACGTT
siNC	mouse	UUCUCCGAACGUGUCACGUTT	ACGUGACACGUUCGGAGAATT

This table lists the nucleotide sequences of the small interfering RNAs (siRNAs) used in this study. Each siRNA includes the sense and antisense strands, along with the target gene information. All sequences were synthesized and purified by standard methods and validated prior to experimental use.

**Table 2:** Sequence of the mRNAs.

Name	Species	Sequence
Ptbp1-Flag	mouse	AGGAGAAUAAACUAGUAUUCUUCUGGUCCCCACAGACUCAGAGAGAACCCGCGGCCGCGCCACCAUGGACGGCAUCGUCCCAGACAUAGCAGUCGGUACAAAGCGGGGAUCCGACGAGCUCUUCUCCACGUGUGUCAGCAACGGCCCCUUCAUCAUGAGCAGCUCUGCCUCAGCAGCCAAUGGAAACGAUAGCAAGAAGUUCAAAGGUGACAACAGGAGCGCAGGAGUCCCUUCCAGAGUCAUCCAUGUCAGAAAGCUGCCCAGCGAUGUCACUGAGGGCGAGGUCAUCUCCCUAGGGCUGCCCUUUGGAAAGGUUACCAACCUUCUCAUGCUGAAGGGGAAGAACCAGGCCUUCAUUGAGAUGAACACAGAGGAGGCUGCCAACACUAUGGUUAACUACUAUACAUCGGUGGCGCCAGUGCUUCGUGGACAGCCCAUCUACAUCCAGUUCUCCAACCACAAAGAGCUCAAGACCGACAGCUCGCCCAACCAGGCACGUGCCCAGGCAGCCCUGCAGGCUGUAAACUCCGUCCAGUCUGGAAACCUGGCCUUGGCAGCGUCCGCUGCUGCCGUGGAUGCAGGAAUGGCAAUGGCAGGGCAGAGCCCAGUGCUCAGGAUCAUUGUGGAAAACCUUUUCUACCCAGUGACCCUGGACGUGCUGCACCAGAUCUUCUCUAAGUUUGGCACCGUCCUGAAGAUCAUCACGUUCACCAAGAACAACCAGUUCCAGGCGCUGCUGCAGUAUGCUGACCCUGUGAGCGCCCAGCAUGCCAAGCUGUCCCUGGAUGGCCAGAACAUCUACAACGCCUGCUGCACGCUGCGCAUCGACUUCUCCAAGCUCACCAGUCUCAAUGUCAAGUACAACAAUGAUAAGAGCAGAGACUACACUCGACCUGACCUGCCCUCUGGAGACAGCCAGCCUUCACUAGACCAGACCAUGGCAGCAGCCUUUGGUGCGCCCGGCAUAAUGUCAGCCUCUCCGUAUGCAGGAGCCGGGUUCCCUCCCACCUUUGCCAUCCCUCAGGCCGCAGGCCUCUCUGUCCCUAAUGUCCAUGGAGCCUUGGCCCCCCUGGCCAUCCCGUCUGCUGCUGCUGCUGCUGCGGCCAGCCGCAUUGCCAUCCCAGGGUUGGCAGGUGCUGGGAAUUCUGUCCUUUUGGUCAGCAAUCUGAACCCUGAGAGAGUCACACCCCAAAGCCUCUUUAUUCUCUUCGGCGUCUACGGUGAUGUGCAGCGGGUGAAGAUCCUGUUCAAUAAGAAGGAGAACGCACUUGUGCAGAUGGCAGACGGCAGCCAGGCCCAGCUGGCCAUGAGCCACCUGAACGGGCACAAGCUGCACGGGAAGUCAGUGCGCAUUACACUGUCCAAGCAUCAGAGUGUGCAGCUGCCUCGGGAGGGUCAGGAGGACCAGGGCCUGACCAAGGACUAUGGCAGCUCCCCGCUGCACCGCUUCAAGAAACCAGGCUCCAAGAACUUCCAGAACAUCUUUCCACCCUCAGCUACCCUGCACCUCUCCAACAUCCCGCCCUCUGUGUCAGAGGACGACCUCAAGAGCCUCUUCUCCAGCAACGGUGGUGUGGUCAAAGGCUUCAAGUUCUUCCAGAAGGACCGCAAGAUGGCACUGAUCCAGAUGGGCUCUGUGGAGGAGGCUGUGCAGGCGCUGAUUGAACUGCACAACCAUGACCUGGGCGAGAACCACCACCUGCGAGUGUCCUUUUCCAAGUCCACCAUCGGGUCCGGAUCGGGUGAUUACAAGGAUGACGACGAUAAGUAGCUCGAGCUGGUACUGCAUGCACGCAAUGCUAGCUGCCCCUUUCCCGUCCUGGGUACCCCGAGUCUCCCCCGACCUCGGGUCCCAGGUAUGCUCCCACCUCCACCUGCCCCACUCACCACCUCUGCUAGUUCCAGACACCUCCCAAGCACGCAGCAAUGCAGCUCAAAACGCUUAGCCUAGCCACACCCCCACGGGAAACAGCAGUGAUUAACCUUUAGCAAUAAACGAAAGUUUAACUAAGCUAUACUAACCCCAGGGUUGGUCAAUUUCGUGCCAGCCACACCCUGGAGCUAGCAAAAAAAAAAAAAAAAAAAAAAAAAAAAAAAAAAAAAAAAAAAAAAAAAAAAAAAAAAAAAAAAAAAAAAAAAAAAAAAAAAAAAAAAAAAAAAAAAAAA
Ptbp1-K258/452R-Flag	mouse	AGGAGAAUAAACUAGUAUUCUUCUGGUCCCCACAGACUCAGAGAGAACCCGCGGCCGCGCCACCAUGGACGGCAUCGUCCCAGACAUAGCAGUCGGUACAAAGCGGGGAUCCGACGAGCUCUUCUCCACGUGUGUCAGCAACGGCCCCUUCAUCAUGAGCAGCUCUGCCUCAGCAGCCAAUGGAAACGAUAGCAAGAAGUUCAAAGGUGACAACAGGAGCGCAGGAGUCCCUUCCAGAGUCAUCCAUGUCAGAAAGCUGCCCAGCGAUGUCACUGAGGGCGAGGUCAUCUCCCUAGGGCUGCCCUUUGGAAAGGUUACCAACCUUCUCAUGCUGAAGGGGAAGAACCAGGCCUUCAUUGAGAUGAACACAGAGGAGGCUGCCAACACUAUGGUUAACUACUAUACAUCGGUGGCGCCAGUGCUUCGUGGACAGCCCAUCUACAUCCAGUUCUCCAACCACAAAGAGCUCAAGACCGACAGCUCGCCCAACCAGGCACGUGCCCAGGCAGCCCUGCAGGCUGUAAACUCCGUCCAGUCUGGAAACCUGGCCUUGGCAGCGUCCGCUGCUGCCGUGGAUGCAGGAAUGGCAAUGGCAGGGCAGAGCCCAGUGCUCAGGAUCAUUGUGGAAAACCUUUUCUACCCAGUGACCCUGGACGUGCUGCACCAGAUCUUCUCUAAGUUUGGCACCGUCCUGAAGAUCAUCACGUUCACCAAGAACAACCAGUUCCAGGCGCUGCUGCAGUAUGCUGACCCUGUGAGCGCCCAGCAUGCCAAGCUGUCCCUGGAUGGCCAGAACAUCUACAACGCCUGCUGCACGCUGCGCAUCGACUUCUCCAGGCUCACCAGUCUCAAUGUCAAGUACAACAAUGAUAAGAGCAGAGACUACACUCGACCUGACCUGCCCUCUGGAGACAGCCAGCCUUCACUAGACCAGACCAUGGCAGCAGCCUUUGGUGCGCCCGGCAUAAUGUCAGCCUCUCCGUAUGCAGGAGCCGGGUUCCCUCCCACCUUUGCCAUCCCUCAGGCCGCAGGCCUCUCUGUCCCUAAUGUCCAUGGAGCCUUGGCCCCCCUGGCCAUCCCGUCUGCUGCUGCUGCUGCUGCGGCCAGCCGCAUUGCCAUCCCAGGGUUGGCAGGUGCUGGGAAUUCUGUCCUUUUGGUCAGCAAUCUGAACCCUGAGAGAGUCACACCCCAAAGCCUCUUUAUUCUCUUCGGCGUCUACGGUGAUGUGCAGCGGGUGAAGAUCCUGUUCAAUAAGAAGGAGAACGCACUUGUGCAGAUGGCAGACGGCAGCCAGGCCCAGCUGGCCAUGAGCCACCUGAACGGGCACAAGCUGCACGGGAAGUCAGUGCGCAUUACACUGUCCAAGCAUCAGAGUGUGCAGCUGCCUCGGGAGGGUCAGGAGGACCAGGGCCUGACCAGGGACUAUGGCAGCUCCCCGCUGCACCGCUUCAAGAAACCAGGCUCCAAGAACUUCCAGAACAUCUUUCCACCCUCAGCUACCCUGCACCUCUCCAACAUCCCGCCCUCUGUGUCAGAGGACGACCUCAAGAGCCUCUUCUCCAGCAACGGUGGUGUGGUCAAAGGCUUCAAGUUCUUCCAGAAGGACCGCAAGAUGGCACUGAUCCAGAUGGGCUCUGUGGAGGAGGCUGUGCAGGCGCUGAUUGAACUGCACAACCAUGACCUGGGCGAGAACCACCACCUGCGAGUGUCCUUUUCCAAGUCCACCAUCGGGUCCGGAUCGGGUGAUUACAAGGAUGACGACGAUAAGUAGCUCGAGCUGGUACUGCAUGCACGCAAUGCUAGCUGCCCCUUUCCCGUCCUGGGUACCCCGAGUCUCCCCCGACCUCGGGUCCCAGGUAUGCUCCCACCUCCACCUGCCCCACUCACCACCUCUGCUAGUUCCAGACACCUCCCAAGCACGCAGCAAUGCAGCUCAAAACGCUUAGCCUAGCCACACCCCCACGGGAAACAGCAGUGAUUAACCUUUAGCAAUAAACGAAAGUUUAACUAAGCUAUACUAACCCCAGGGUUGGUCAAUUUCGUGCCAGCCACACCCUGGAGCUAGCAAAAAAAAAAAAAAAAAAAAAAAAAAAAAAAAAAAAAAAAAAAAAAAAAAAAAAAAAAAAAAAAAAAAAAAAAAAAAAAAAAAAAAAAAAAAAAAAAAAA

This table provides the nucleotide sequences of the mRNAs analyzed in this study. For each mRNA, the full-length sequence is listed, along with the corresponding gene name and annotation. All sequences were verified against reference databases prior to experimental analysis.

### Measurement of cell viability

Cell viability of BV2 cells was assessed using the Cell Counting Kit-8 (CCK-8) assay (APExBIO Technology LLC, K1018). Briefly, cells were seeded into 96-well plates at a density of 2000 cells per well in 100 μl of complete medium and subjected to the designated experimental treatments. After treatment, 10 μl of CCK-8 reagent was added to each well, and the plates were incubated at 37 °C for 2 h. Absorbance was subsequently measured at a wavelength of 450 nm using a microplate reader to quantify viable cells.

### Measurement of lactate levels

BV2 cells were homogenized in ice-cold lysis buffer and sonicated on ice using a 300 W output (3 s pulse-on, 7 s pulse-off) for a total duration of 3 min. The homogenates were then centrifuged at 12,000 × g for 10 min at 4 °C. The resulting supernatant was collected, and lactate concentration was quantified using a commercial lactate colorimetric assay kit (Elabscience Biotechnology Co., Ltd, E-BC-K044-M) in accordance with the manufacturer’s protocol. Absorbance was measured at 570 nm with a microplate reader to determine lactate levels. All experiments were performed in triplicate to ensure reproducibility.

### Immunofluorescence staining

Cells were seeded onto coverslips (Biosharp) in a 24-well plate. Following OGD/R treatment, the culture medium was aspirated and cells were washed three times with PBS. Subsequently, cells were fixed with 4% paraformaldehyde for 10 min at room temperature, permeabilized with 0.1% Triton X-100 for 10 minutes, and blocked with 5% bovine serum albumin (BSA) for 30 minutes. After blocking, cells were incubated overnight at 4 °C with specific primary antibodies, followed by a 1-hour incubation at room temperature in the dark with the corresponding fluorescently conjugated secondary antibodies and DAPI. Finally, the coverslips were mounted onto glass slides using an anti-fade mounting medium and imaged with a Leica confocal microscope. Fluorescence intensity was quantified with ImageJ software. The primary and secondary antibodies used are listed as follows: anti-GFAP (GeneTex, GTX85454, 1:400); anti-IBA1 (Abcam, ab5076, 1:200); anti-Pan-Kla (PTM-BIO, PTM-1401, 1:100); anti-FLAG (Vazyme, RA1003-01, 1:100); Donkey anti-Rabbit Alexa Fluor™ 488 (Invitrogen, A-21206, 1:1000); Donkey anti-Mouse Alexa Fluor™ 568 (Invitrogen, A10037, 1:1000); Goat anti-Chicken Alexa Fluor™ 647 (Invitrogen, A-21449, 1:1000); Donkey anti-Goat Alexa Fluor™ 633 (Invitrogen, A21082, 1:1000).

### Protein extraction and western blot analysis

BV2 cells were lysed on ice for 30 min using RIPA buffer (Beyotime, P0013B) supplemented with protease and phosphatase inhibitors ((Beyotime, P1005). The lysates were then centrifuged at 12,000 rpm for 10 min at 4 °C, and the supernatant was collected for protein quantification using a BCA assay ((Beyotime, P0012S). Proteins were denatured in SDS loading buffer at 100 °C for 10 min and separated by SDS-PAGE. Proteins were then electrophoretically transferred to a polyvinylidene fluoride (PVDF) membrane (Millipore). The membrane was blocked with 5% non-fat milk in TBST for 2 h at room temperature, followed by incubation with specific primary antibodies overnight at 4 °C with gentle shaking. After washing three times with TBST, the membrane was incubated with an HRP-conjugated secondary antibody for 1 h at room temperature. Protein bands were visualized using a ChemiDoc XRS+ imaging system (Bio-Rad Laboratories). All Western blot signals were quantified by ImageJ software and normalized to β-actin levels, which served as the internal loading control for each sample. Relative protein expression levels were then calculated as the ratio of the target protein signal to the corresponding β-actin signal. The primary antibodies used are listed as follows: anti-Pan-Kla (PTM-BIO, PTM-1401, 1:1000); anti-BCL-2 (Proteintech, 26593-1-AP, 1:1000); anti-BAX (ABclonal, A0207, 1:1000); anti-Caspase-3 (Cell Signaling Technology, 9662S, 1:1000); anti-cleaved Caspase-3 (Cell Signaling Technology, 9661S, 1:1000); anti-PTBP1 (Cell Signaling Technology, 57246S, 1:1000); anti-FLAG (Vazyme, RA1003-01, 1:1000); anti-USP18 (Cell Signaling Technology, 53229 T, 1:1000); anti-FTO (ABclonal, A1438, 1:1000); anti-SIRT1 (Cell Signaling Technology, 9475 T, 1:1000); anti-SIRT2 (ABclonal, A12575, 1:1000); anti-SIRT3 (Cell Signaling Technology, 5490S, 1:1000); anti-Ubiquitin (Proteintech, 10201-2-AP, 1:1000); anti-β-ACTIN (Proteintech, 20536-1-AP, 1:5000). The uncropped original western blots are provided in the Supplemental materials.

### Co-immunoprecipitaion (Co-IP)

BV2 cells were lysed with IP lysis buffer containing protease and phosphatase inhibitors using Classic Magnetic Bead-Based IP/Co-IP Kit (Thermo Fisher Scientific, 88804). The lysates were centrifuged at 10,000 rpm for 10 min at 4 °C, and the supernatant was collected. For each Co-IP assay, the cell lysate was incubated with a specific primary antibody overnight at 4 °C with gentle rotation, followed by incubation with protein A/G agarose beads for 1 h at room temperature. After extensive washing, the immunoprecipitated complexes were eluted and subjected to western blot analysis for target protein detection.

### Detection of PTBP1 lactylation

PTBP1 lactylation was assessed by immunoprecipitation (IP) followed by immunoblotting with a pan-Kla antibody. Briefly, cell lysates were incubated overnight at 4 °C with anti-PTBP1 antibody (Cell Signaling Technology, 57246S), followed by incubation with Protein A/G agarose beads for 2 h. Immunoprecipitated complexes were analyzed by SDS-PAGE and immunoblotted with pan-Kla antibody (PTM-BIO, PTM-1401; 1:1000). Membranes were then stripped and re-probed with anti-PTBP1 antibody to confirm IP efficiency.

### RNA immunoprecipitation (RIP)

RNA immunoprecipitation (RIP) was performed using the BeyoRIP™ RIP Assay Kit (Beyotime, P1805S) following the manufacturer’s protocol. Cells were washed twice with ice-cold PBS and lysed in RIP lysis buffer supplemented with protease and RNase inhibitors for 10 min on ice. Lysates were centrifuged at 12,000 × g for 10 min at 4 °C, and the supernatants were collected for immunoprecipitation. Protein A/G magnetic beads were washed twice with RIP wash buffer and incubated with either anti-PTBP1 antibody or non‑specific rabbit IgG for 1 h at room temperature with rotation. The pre‑cleared cell lysate was then added to the antibody‑bead complexes and incubated overnight at 4 °C with gentle rotation to capture RNA-protein complexes. On the following day, the beads were washed three times with RIP wash buffer to remove non‑specifically bound proteins. Immunoprecipitated complexes were resuspended in protease K digestion buffer and incubated at 55 °C for 30 min to reverse crosslinks and digest proteins. RNA was subsequently extracted from the immunoprecipitates using RNA purification columns (Beyotime, R0027). Enrichment of target RNAs was assessed by real‑time quantitative PCR (qPCR). RNA abundance was quantified as a percentage of input (%input) and presented as fold enrichment relative to the IgG control. All experiments were performed in three independent biological replicates.

### Proteomic profiling of lactylation using liquid chromatography-tandem mass spectrometry (LC-MS/MS)

BV2 cells were cultured under either OGD/R or normal conditions. To enrich lysine lactylation (Kla)-modified peptide segments, protein samples were extracted and digested with trypsin in 100 mM TEAB buffer. The digested peptides were desalted using a C18 column, washed with a cleaning solution (0.1% formic acid, 3% acetonitrile), and eluted with an appropriate amount of elution buffer (0.1% formic acid, 70% acetonitrile). The eluate was collected and lyophilized, redissolved in binding buffer, and incubated with anti-lactylation antibody-conjugated magnetic beads at room temperature for 2 h with gentle inversion. After incubation, the beads were washed and bound Kla peptides were eluted. For LC-MS/MS analysis, peptides were resuspended in mobile phase A (0.1% formic acid in water) and separated using a UHPLC system (Vanquish Neo Nano, Thermo Scientific) coupled to an Orbitrap Astral mass spectrometer equipped with an Easy Spray (ESI) ion source. Chromatography was performed using a gradient of mobile phase B (0.1% formic acid in 80% acetonitrile). Data-independent acquisition (DIA) was used for mass spectrometry detection.

The raw data were processed using Spectronaut software for library-based DIA analysis, retaining peptide-spectrum matches (PSMs) with a confidence level exceeding 99% and proteins with at least one unique peptide. False discovery rate (FDR) was controlled at less than 1% at both peptide and protein levels. Differentially expressed proteins (DEPs) were identified using a t-test (*p* < 0.05, |log₂FC | > 1.0 [fold change, FC]) and further subjected to functional annotation via InterProScan software integrating Pfam, PRINTS, ProDom, SMART, ProSite, and PANTHER databases. Protein family and pathway analyses were carried out using COG and KEGG databases, and protein-protein interaction networks were predicted using STRING DB software. Visualization included volcano plots, hierarchical clustering heatmaps, and enrichment analyses of GO terms, InterPro domains, and KEGG pathways.

### Bulk RNA-sequencing and transcriptome analysis

RNA integrity and quantity were rigorously assessed using an Agilent 2100 Bioanalyzer, and total RNA was used as input for cDNA library preparation. Polyadenylated mRNA was enriched using oligo(dT) magnetic beads, followed by cDNA synthesis, fragmentation, and adapter ligation. cDNA libraries were size-selected for fragments ranging from 370-420 bp using AMPure XP beads, amplified by PCR, and again purified with AMPure XP beads. Final libraries were quantified with a Qubit 2.0 Fluorometer, and size distribution was assessed with the Agilent 2100 Bioanalyzer. Equimolar pools of libraries were constructed based on accurate quantification and subjected to Illumina sequencing to generate 150 bp paired-end reads.

Raw sequencing reads in FASTQ format, containing nucleotide sequences and their corresponding quality scores, were subjected to rigorous quality control by removing adapter sequences, reads containing ambiguous bases, and low-quality reads where over 50% of bases had a Qphred score ≤ 20. The resulting high-quality clean data were further evaluated by calculating Q20, Q30 scores, and GC content. Differential gene expression (DEG) analysis between comparative groups was conducted using DESeq2 software (v1.20.0), and functional enrichment analyses of GO terms and KEGG pathways for the DEGs were performed using the clusterProfiler package (v3.8.1).

### Statistical analysis

Statistical analysis was conducted using GraphPad Prism software. For comparisons between two groups, unpaired two-tailed Student’s t-tests are applied, whereas one-way ANOVA followed by Turkey’s post hoc test is used for multi-group comparisons, unless otherwise noted. All quantitative data, derived from at least three independent biological replicates, are presented as mean ± standard deviation (SD). Sample size for each experiment is stated in the related figure legend. A p-value of less than 0.05 was considered statistically significant.

## Results

### Cerebral hypoxia-ischemia induces protein lactylation and cellular apoptosis in microglia

**Fig. 1:**
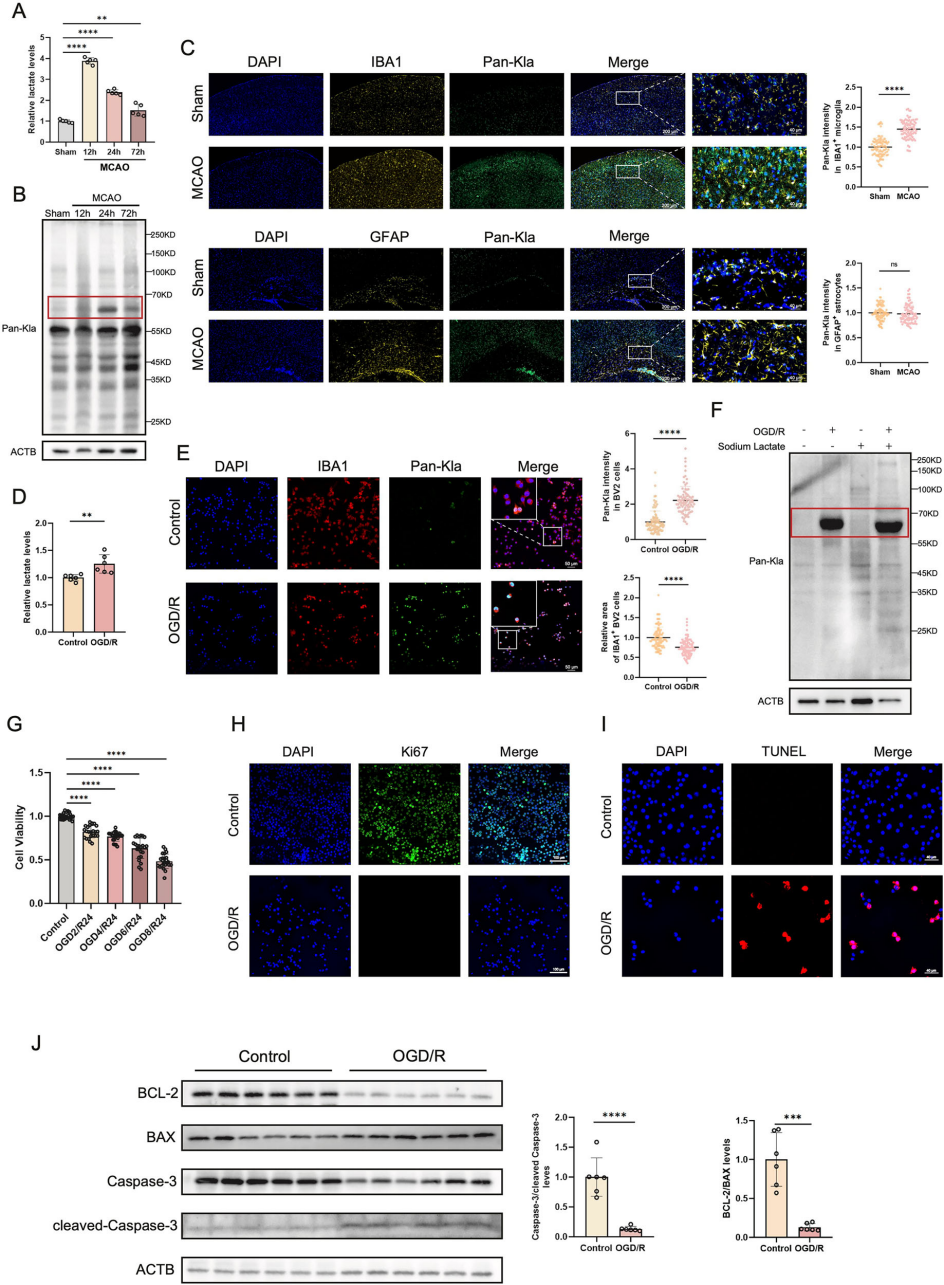
Hypoxic-ischemic conditions induce cellular apoptosis and protein lactylation. **A** Lactate levels in the cerebral cortex of sham-operated and MCAO mice at 12, 24, and 72 h after reperfusion (*N* = 5 per group). **B** Western blot analysis of Pan-Kla in cerebral cortex of mice subjected to MCAO, compared to sham-operated controls, at 12, 24, and 72 h post-surgery. **C** GFAP (astrocyte marker)/IBA-1 (microglial marker) and Pan-Kla double-stained confocal images of cerebral cortex in sham-operated and MCAO mice at 24 h after reperfusion (*N* = 3 mice per group, *n* = 10 randomly selected fields per section across 3 sections per animal). Resident microglia display a ramified morphology with fine, branched processes and relatively small, irregularly shaped cell bodies, and are evenly distributed within the neural parenchyma. Quantification was performed accordingly to ensure specific assessment of microglial responses. Scale bars, 200 μm (lower-resolution images) or 40 μm (higher-resolution images). **D** Lactate levels in OGD/R-treated BV2 cells. **E** Immunofluorescence detection of Pan-Kla levels in OGD/R-treated BV2 cells (*N* = 6 independent passages cultured and treated on separate days; *n* = 15 randomly selected fields per biological replicate). Scale bar, 50 μm. **F** Western blot analysis of Pan-Kla in OGD/R-treated BV2 cells with or without additional sodium lactate. **G** Cell viability of BV2 cells exposed to 2, 4, 6, or 8 h OGD followed by 24 h reoxygenation. **H** Ki67 staining in BV2 cells under control and OGD/R conditions. Scale bar, 100 μm. **I** TUNEL staining in BV2 cells under control and OGD/R conditions. Scale bar, 40 μm. **J** Western blot analysis showing the changes in the levels of BCL-2, BAX, Caspase-3 and cleaved Caspase-3 in OGD/R-treated BV2 cells. For comparisons between two groups, unpaired two-tailed Student’s t-tests are applied, whereas one-way ANOVA followed by Turkey’s post hoc test is used for multi-group comparisons. All quantitative data, derived from at least three independent biological replicates, are presented as mean ± standard deviation (SD). **p  < 0.01, ***p < 0.001, ****p < 0.0001, ns: no significance.

Cerebral hypoxia-ischemia triggers a pathological cascade involving severe dysregulation of cellular energy metabolism, primarily marked by aberrant lactate accumulation due to accelerated anaerobic glycolysis [28, 29], we therefore examined lactate levels in the cerebral cortex of mice following middle cerebral artery occlusion (MCAO). Colorimetric assays revealed a significant increase in lactate levels in MCAO mice compared to the sham controls (Sham), with a peak at 12 hours post-reperfusion and subsequent decline at 24 and 72 h (Fig. 1A). Given that lactate is the essential substrate for protein lactylation [30], we hypothesize that cerebral ischemia-induced accumulation in lactate operates as a global metabolic rheostat that dynamically reprograms the pan-proteomic lactylation landscape. Western blot analysis demonstrated elevated pan-lysine lactylation (Pan-Kla), particularly within the 55-70 kDa range, at 24 hours post-reperfusion in MCAO mice (Fig. 1B). To examine the cell-type specificity of lactylation, and considering the central roles of astrocytes and microglia as metabolically adaptable immune cells in the CNS, we performed immunofluorescence co-staining of Pan-Kla along with cell-specific markers (GFAP for astrocytes, IBA1 for microglia). At 24 h post-reperfusion in MCAO mice, microglia exhibited a pronounced increase in Pan-Kla signal intensity in the cerebral cortex, whereas no significant alterations of Pan-Kla intensity were observed in astrocyte (Fig. 1C).

Consistent with the elevated lactate levels observed in microglia of MCAO mice, the murine microglial BV2 cell line subjected to oxygen-glucose deprivation/reoxygenation (OGD/R) also exhibited a significant increase in intracellular lactate levels relative to normal conditions (Fig. 1D). Immunofluorescence and Western blot analyses demonstrated a pronounced upregulation of Pan-Kla in OGD/R-treated cells, manifesting as enhanced immunofluorescence signals and increased immunublot signals (specifically within the 55-70 kDa range) compared to normoxic controls (Fig. 1E, F). The administration of exogenous sodium lactate further augmented Pan-Kla levels within the 55–70 kDa range, indicating a lactate-driven regulatory mechanism under OGD/R conditions (Fig. 1F). In addition, immunofluorescence analysis revealed an inverse correlation between Pan-Kla intensity and the cellular area of IBA1⁺ BV2 microglia under OGD/R stress. While the precise mechanism underlying this morphological change remains to be fully elucidated, one possibility is that elevated lactylation may be associated with transcriptional programs that could influence cellular shrinkage, a feature reminiscent of apoptotic volume decrease observed during programmed cell death (Fig. 1E).

To investigate OGD/R-induced apoptosis in BV2 cells, cellular viability was assessed using the CCK-8 assay following 24 hours of reoxygenation after oxygen-glucose deprivation (OGD) for 2, 4, 6, or 8 hours, which demonstrated a progressive decline in survival with extended OGD duration (Fig. 1G). Moreover, OGD/R led to a significant reduction in cell proliferation, reflected by a decrease in Ki67-positive cell populations (Fig. 1H), and a marked increase in apoptosis as assessed by TUNEL staining (Fig. 1I).Western blot analysis further revealed a significant reduction in the anti-apoptotic to pro-apoptotic protein (BCL-2/BAX) ratio and enhanced cleavage of Caspase-3 in BV2 cells following an 8-hour OGD and a 24-hour reoxygenation (Fig. 1J). The observed correlation between increased protein lactylation and reduced cell survival raises the hypothesis that lactate-induced lysine lactylation may play a functional role in the initiation and execution of apoptosis.

Furthermore, we validated these findings using the human microglial clone 3 (HMC3) cell line, a well-characterized in vitro model for studying human microglial biology. Following OGD/R treatment, HMC3 cells exhibited increased global lactylation levels (Supplemental Fig. 1A). Additionally, OGD/R induced a significant reduction in HMC3 cell proliferation, as evidenced by a decreased proportion of Ki67-positive cells (Supplemental Fig. 1B), and significantly promoted apoptosis which confirmed by Western blot analysis and TUNEL staining (Supplemental Fig. 1C, D).

Collectively, these observations demonstrate an extensive elevation in both lactylation and apoptotic activity in microglia under OGD/R conditions, indicative of a model in which lactate accumulation may be a driver for a lactylation-mediated apoptotic program in hypoxic-ischemic injury.

### Global lactylome profiling identifies PTBP1 as a critical hyper-lactylated protein in OGD/R-treated microglia

**Fig. 2:**
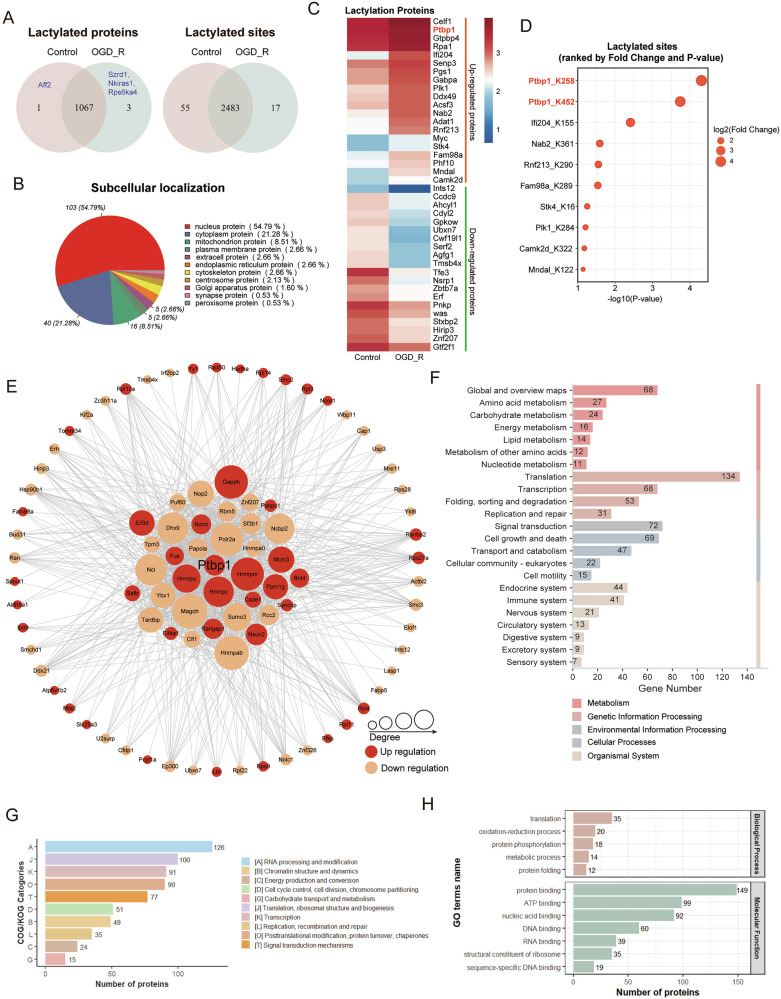
Lactylome analysis reveals PTBP1 hyperlactylation in OGD/R-treated microglia. **A** Venn diagrams illustrating the overlap and unique identification of lactylated proteins and sites between control and OGD/R-treated BV2 cells. **B** Subcellular localization of lactylated proteins. **C** Heatmap of the top 20 most significantly up- and down-regulated lactylated proteins between control and OGD/R conditions. **D** Dotplot of the top 10 most significantly upregulated lactylated sites under OGD/R. **E** Protein-protein interaction network of differentially lactylated proteins, constructed using the STRING database. The node size corresponds to the relative fold-change in lactylation levels under OGD/R. **F** The KEGG enrichment analysis of differentially lactylated proteins. **G** The COG/KOG enrichment analysis of differentially lactylated proteins. **H** The GO enrichment analysis of differentially lactylated proteins.

Lactylation is increasingly recognized as a pivotal post-translational modification that mediates alterations in protein conformation and diverse cellular functions [31, 32]. To comprehensively characterize the lactylation landscape in microglia under hypoxic-ischemic conditions, we treated BV2 cells with an 8-hour OGD followed by a 24-hour reoxygenation and performed data-dependent acquisition (DDA) mass spectrometry-based proteomic analysis. Global lactylome profiling identified 2555 lactylated sites across 1071 proteins in OGD/R-treated BV2 cells (Fig. 2A). Differential analysis revealed 352 significantly dysregulated lactylated sites (*p* < 0.05), comprising 161 hyper-lactylated and 191 hypo-lactylated sites, with a notable enrichment of nuclear-localized proteins (Fig. 2B, Supplemental Fig. 2).

From comparative lactylome analysis of proteins within 55–70 kDa range, we identified polypyrimidine tract-binding protein 1 (PTBP1) (57 kDa; Supplemental File 1) as a high-priority candidate among the top 20 hyper-lactylated and hypo-lactylated proteins (55–70 kDa range) based on its pronounced lactylation remodeling, which was marked by two significantly hyper-lactylated sites at K258 (log₂FC = 4.7, *p* < 0.001) and K452 (log₂FC = 4.3, *p* < 0.001) following OGD/R exposure (Fig. 2C, D). Protein-protein interaction analysis using the Search Tool for the Retrieval of Interacting Genes/Proteins (STRING) database confirmed PTBP1 served as a central hub of the interaction network among lactylated proteins (Fig. 2E). These findings were further supported by functional enrichment analyses (KEGG, KOG/COG, and GO), which revealed significant convergence of lactylated proteins on biological pathways related to transcriptional regulation, RNA processing and modification, and RNA binding (Fig. 2F–H), all processes coherent with PTBP1’s canonical functions. Together, these results identify the RNA-splicing factor PTBP1 as a pivotal highly lactylated protein in OGD/R-treated microglia.

### Lactylation of PTBP1 at Lysines 258 and 452 drives microglial apoptosis under hypoxic-ischemic stress

**Fig. 3:**
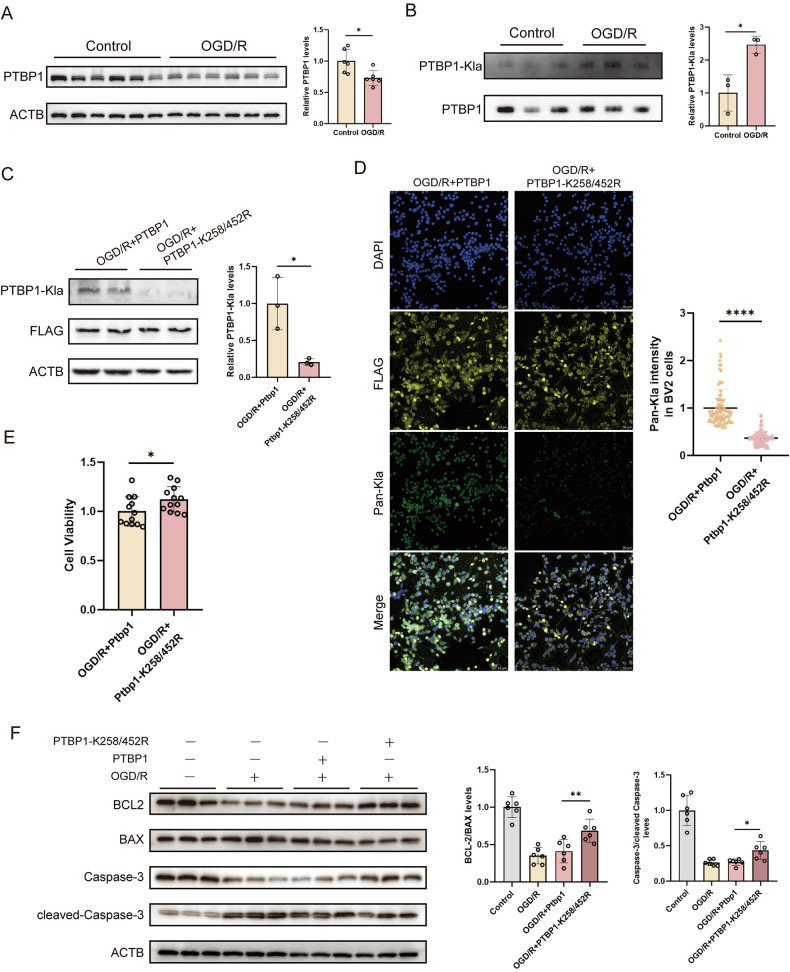
Site-specific lactylation of PTBP1 triggers microglial apoptosis under hypoxic-ischemic stress. **A** Western blot analysis of PTBP1 in OGD/R-treated BV2 cells. **B** Lactylation of PTBP1 in BV2 cells under OGD/R. **C** Assessment of lactylated PTBP1 in OGD/R-treated BV2 cells after overexpression of FLAG-tagged PTBP1 or its mutant PTBP1-K258/452 R. **D** Immunofluorescence detection of Pan-Kla levels in BV2 cells with FLAG-tagged PTBP1 or PTBP1-K258/452 R overexpression under OGD/R (*N* = 6 independent passages; *n* = 15 randomly selected fields per biological replicate). Scale bars, 50 μm. **E** Cell viability of BV2 cells with FLAG-tagged PTBP1 or PTBP1-K258/452 R overexpression under OGD/R. **F** Western blot analysis showing the changes in the levels of BCL-2, BAX, Caspase-3, and cleaved Caspase-3 in BV2 cells under control conditions or subjected OGD/R, in the presence of overexpressed FLAG-tagged PTBP1 or PTBP1-K258/452 R under OGD/R. For comparisons between two groups, unpaired two-tailed Student’s t-tests are applied, whereas one-way ANOVA followed by Turkey’s post hoc test is used for multi-group comparisons. All quantitative data, derived from at least three independent biological replicates, are presented as mean ± SD. *p < 0.05, **p < 0.01, ****p < 0.0001.

Given the established role of PTBP1 in transcript processing and cell apoptosis [33, 34], we hypothesized its hyper-lactylation could functionally link to apoptotic pathways. To define its role in OGD/R-induced microglial apoptosis, we first assessed PTBP1 protein and lactylation levels, revealing a decreased in total PTBP1 expression but a significant elevation in its lactylation modification (Fig. 3A, B). PTBP1 is known to promote cell proliferation [33, 35, 36]; however, the observed reduction in its total abundance coupled with enhanced lactylation suggests that this modification might compromise its naive function and promote a shift toward apoptosis. To test this, we generated a PTBP1 K258R/K452R double mutant to mimic a constitutively delactylated state, guided by high-confidence MS/MS spectral data that identified these sites (K258 and K452) through characteristic lactylation-specific fragment ions (Supplemental Fig. 3).

Transfection of OGD/R-treated BV2 cells with mRNAs encoding FLAG-tagged wile-type PTBP1 or the lactylation-deficient PTBP1 K258R/K452R mutant resulted in comparable protein overexpression, while lactylation signals were significantly attenuated in cells expressing the PTBP1 lysine-mutant variant (Fig. 3C). This reduction in lactylation levels of the PTBP1 K258R/K452R mutant was further validated by immunofluorescence analysis, displaying downregulated Pan-Kla intensity in FLAG-tagged BV2 cells (Fig. 3D). Functionally, BV2 cells expressing the lactylation-deficient mutant exhibited increased cell viability (Fig. 3E), an elevated anti-apoptotic BCL-2/BAX ratio, and diminished Caspase-3 cleavage compared to wild-type PTBP1-expression controls (Fig. 3F), collectively indicating a strong anti-apoptotic effect. This cytoprotective phenotype upon disruption of PTBP1 lactylation demonstrates that site-specific lactylation at K258 and K452 is a critical regulatory mechanism through which PTBP1 promotes microglial apoptosis under OGD/R conditions.

### Lactylated PTBP1 mediates microglial apoptosis via suppression of USP18 following OGD/R injury

**Fig. 4:**
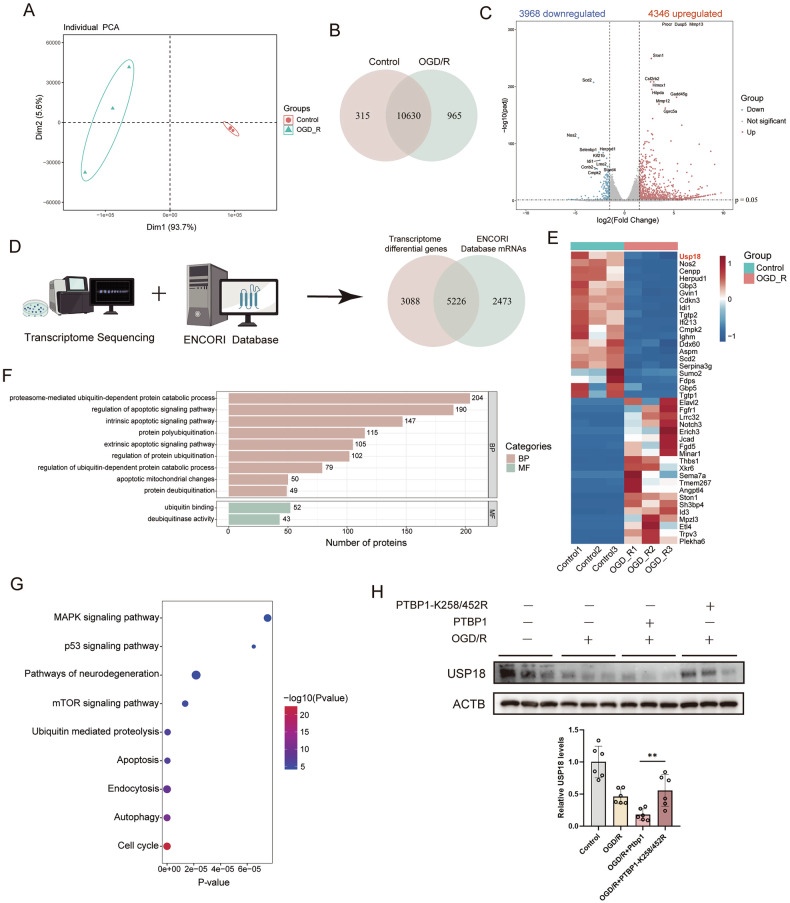
Lactylated PTBP1 suppresses USP18 expression in OGD/R-treated BV2 cells. **A** PCA of control and OGD/R-treated BV2 cells. **B** Venn diagram illustrating the overlap and unique identification of genes between control and OGD/R groups. **C** Volcano plot of differentially expressed genes between control and OGD/R groups. **D** Flowchart illustrating the screening of Ptbp1-interacting mRNAs that are differentially expressed between control and OGD/R groups. **E** Heatmap of the top 20 most significantly up- and down-regulated genes between control and OGD/R groups. **F** The GO enrichment analysis of Ptbp1-interacting mRNAs that are differentially expressed between control and OGD/R groups. **G** The KEGG enrichment analysis of Ptbp1-interacting mRNAs that are differentially expressed between control and OGD/R groups. **H** Western blot analysis of USP18 expression in BV2 cells under control conditions or subjected to OGD/R, in the presence of overexpressed FLAG-tagged PTBP1 or PTBP1-K258/452 R under OGD/R. One-way ANOVA followed by Turkey’s post hoc test is applied. All quantitative data, derived from at least three independent biological replicates, are presented as mean ± SD. **p < 0.01.

To delineate the mechanistic basis by which lactylated PTBP1 regulates the potential apoptotic effectors in OGD/R-treated BV2 microglia, we conducted transcriptome sequencing to identify its downstream targets. Principal component analysis (PCA) revealed robust segregation between control and OGD/R groups, indicating substantial divergence in global gene expression profiles induced by hypoxic-ischemic stress (Fig. 4A). Transcriptomic analysis revealed that a total of 10 945 genes were detected in the control group, and 11 595 genes were detected in the OGD/R-treated group, with 10 630 genes commonly expressed in both conditions (Fig. 4B). Comparative analysis further identified 8 314 differentially expressed genes (DEGs) (*p* < 0.05), comprising 4 346 upregulated and 3 968 downregulated transcripts (Fig. 4C). Integration of these data with the ENCORI Ptbp1-RNA interactome database revealed 5 226 Ptpb1-bound mRNAs that were differentially expressed and functionally implicated in apoptotic regulatory pathways (Fig. 4D). From these candidates, we prioritized ubiquitin-specific peptidase 18 (Usp18), a key deubiquitinating enzyme involved in apoptotic pathways [37, 38], as its notable downregulation under OGD/R conditions (log_2_FC = -2.1) among the most altered Ptbp1-interacting transcripts (Fig. 4E). Functional enrichment analysis of these targets confirmed marked enrichment of apoptosis and deubiquitination-related pathways, supporting a role for Usp18 as a potential apoptotic regulator in OGD/R-treated BV2 microglia (Fig. 4F, G). Consistent with the transcriptomic data, qPCR and Western blot analyses confirmed the reduction of USP18 expression levels in OGD/R-treated BV2 microglia (Fig. 4H; Supplemental Fig. 4A). Conversely, PTBP1 lactylation-deficient mutant (K258R/K452R) restored USP18 protein levels in microglia under OGD/R (Fig. 4H), establishing lactylated PTBP1 as a critical upstream regulator of USP18 expression. These findings demonstrate that lactylated PTBP1 triggers apoptosis in hypoxic-ischemic microglia through suppressing USP18 expression.

To determine whether PTBP1 lactylation directly modulates USP18 mRNA stability, we immunoprecipitated endogenous PTBP1-bound RNA from BV2 cells using a PTBP1-specific antibody. RT-qPCR analysis revealed significant enrichment of Usp18 mRNA in PTBP1 immunoprecipitates compared to IgG controls, confirming a direct physical association between PTBP1 and USP18 transcripts. Notably, this interaction was reduced under OGD/R conditions, suggesting that stress-induced PTBP1 lactylation may modulate its RNA-binding affinity (Supplemental Fig. 4B).

### Lactylated PTBP1 drives apoptotic responses in hypoxic-ischemic microglia through regulating USP18-FTO-SIRT1 signaling axis

**Fig. 5:**
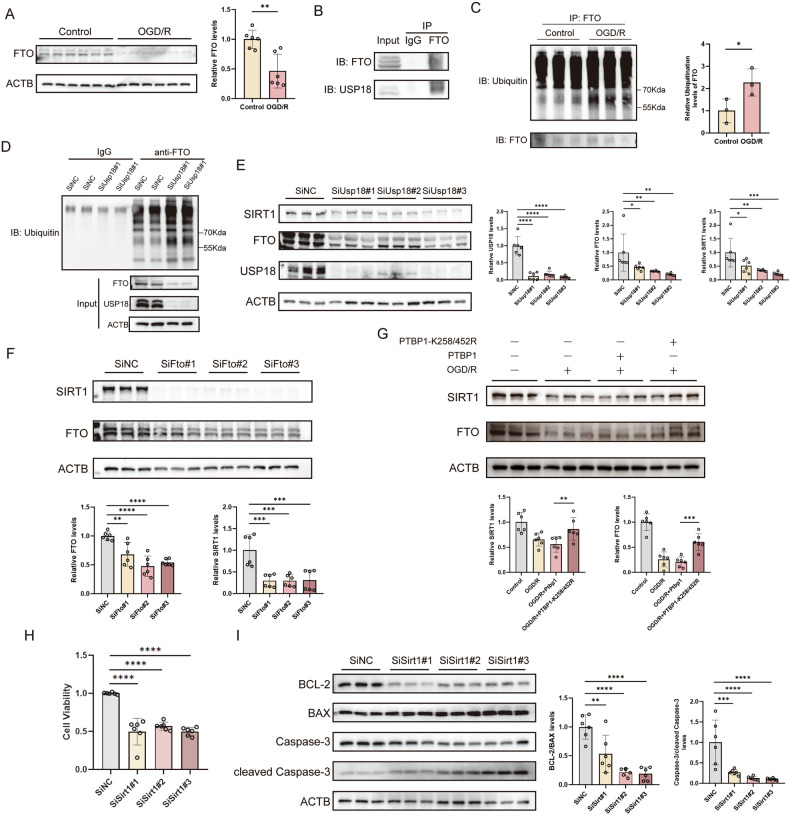
Lactylated PTBP1 drives microglial apoptosis via the USP18-FTO-SIRT1 axis under hypoxic-ischemic stress. **A** Western blot analysis of FTO levels in OGD/R-treated BV2 cells. **B** Co-IP analysis of the interplay between USP18 and FTO proteins. **C** Ubiquitination levels were detected by Western blotting after FTO immunoprecipitation in OGD/R-treated BV2 cells. **D** Ubiquitination levels were detected by Western blotting after USP18 knockdown and FTO immunoprecipitation. Ubiquitination levels were assessed by total cell lysates with an anti-ubiquitin antibody, which detects polyubiquitinated proteins across all molecular weight ranges. The representative blots include the full molecular weight spectrum, and quantification reflects total cellular ubiquitination signals. **E** Western blot analysis of USP18, FTO, and SIRT1 in BV2 cells following USP18 knockdown. **F** Western blot analysis of FTO and SIRT1 in BV2 cells following FTO knockdown. **G** Western blot analysis of FTO and SIRT1 in BV2 cells under control conditions or subjected to OGD/R, in the presence of overexpressed FLAG-tagged PTBP1 or PTBP1-K258/452 R under OGD/R. **H** Cell viability of BV2 cells following SIRT1 knockdown. **I** Western blot analysis of BCL-2, BAX, Caspase-3, and cleaved Caspase-3 levels in BV2 cells following SIRT1 knockdown. For comparisons between two groups, unpaired two-tailed Student’s t-tests are applied, whereas one-way ANOVA followed by Turkey’s post hoc test is used for multi-group comparisons. All quantitative data, derived from at least three independent biological replicates, are presented as mean ± SD. *p < 0.05, **p < 0.01, ***p < 0.001, ****p < 0.0001.

Given previous reports demonstrating that USP18 modulates mitophagy and ferroptosis through deubiquitination of fat mass and obesity-associated protein (FTO) [37, 39], a multifunctional regulator of energy metabolism with broad therapeutic relevance [37, 40, 41], we hypothesized that FTO acts as a critical downstream effector of USP18-mediated cell apoptosis. Analysis of the PhosphoSitePlus database revealed ten conserved ubiquitination sites (K88, K194, K216, etc.) on FTO (Supplemental Fig. 5), promoting us to investigate whether USP18 regulates FTO protein stability to induce microglial apoptosis under OGD/R conditions. First, we detected the expression pattern of FTO in OGD/R-treated BV2 microglia and observed a significant decrease under OGD/R stress (Fig. 5A). In addition, we performed co-immunoprecipitation assays, which demonstrated that FTO specifically co-precipitated with USP18, confirming a direct physical interaction between these proteins (Fig. 5B). Notably, FTO ubiquitination was significantly elevated in OGD/R-treated BV2 cells (Fig. 5C), and USP18 knockdown further amplified FTO ubiquitination levels (Fig. 5D), implicating that reduced USP18 expression promotes ubiquitination-mediated degradation of FTO under hypoxic-ischemic stress.

To determine the downstream apoptotic effectors regulated by the USP18-FTO axis, we examined sirtuin 1 (SIRT1), a critical regulator of cellular stress resistance in OGD/R-treated cells [42, 43]. Post-transcriptional gene silencing of USP18 reduced both FTO and SIRT1 expression (Fig. 5E), and FTO knockdown decreased SIRT1 (Fig. 5F). Conversely, FTO and SIRT1 protein levels were increased in BV2 microglia expressing a lactylation-deficient PTBP1 mutant under OGD/R (Fig. 5G), further supporting that SIRT1 is a downstream effector of the lactylated PTBP1-USP18-FTO regulatory axis. Functional validation confirmed that SIRT1 knockdown in BV2 microglia markedly reduced cell viability (Fig. 5H) and shifted the balance toward apoptosis, as evidenced by a decreased BCL-2/BAX ratio and increased Caspase-3 cleavage (Fig. 5I). Collectively, these results define a coherent lactylated PTBP1-USP18-FTO-SIRT1 signaling axis through which lactylation of PTBP1 promotes microglial apoptosis under hypoxic-ischemic conditions.

### SIRT1 deficiency promotes OGD/R-treated microglial apoptosis by driving a PTBP1 hyper-lactylation-dependent positive feedback loop

**Fig. 6:**
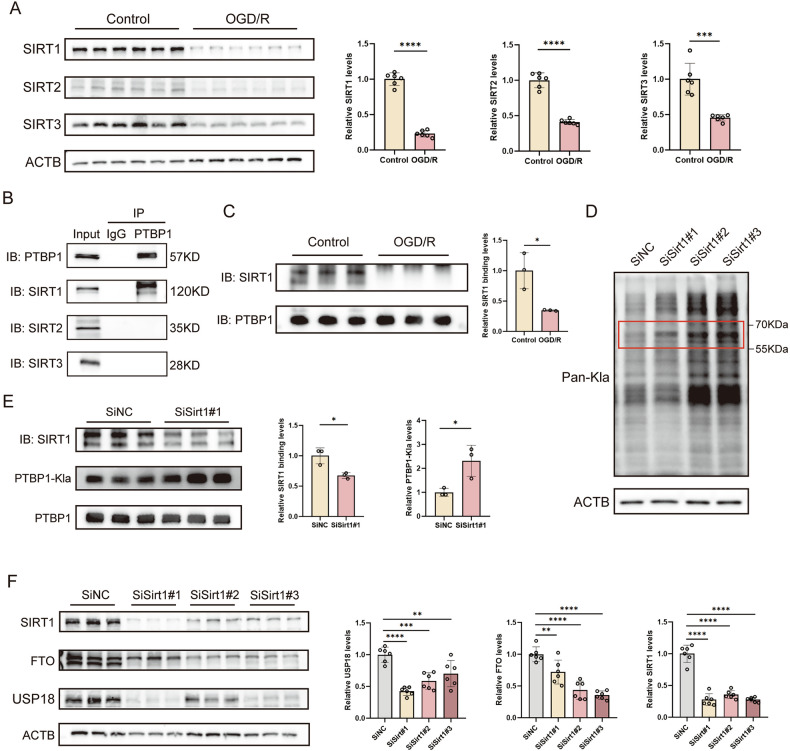
SIRT1 deficiency promotes the lactylation of PTBP1. **A** Western blot analysis of SIRT1, SIRT2, and SIRT3 in BV2 cells under OGD/R. **B** Co-IP analysis of the interplay between PTBP1 and SIRT1/2/3 proteins. **C** Western blot analysis assessing the interaction between SIRT1 and PTBP1 in BV2 cells under OGD/R. **D** Western blot analysis of Pan-Kla in BV2 cells following SIRT1 knockdown. **E** Western blot analysis of PTBP1-Kla levels and the interaction between SIRT1 and PTBP1 in BV2 cells following SIRT1 knockdown. **F** Western blot analysis of USP18, FTO, and SIRT1 in BV2 cells following SIRT1 knockdown. For comparisons between two groups, unpaired two-tailed Student’s t-tests are applied, whereas one-way ANOVA followed by Turkey’s post hoc test is used for multi-group comparisons. All quantitative data, derived from at least three independent biological replicates, are presented as mean ± SD. *p < 0.05, **p < 0.01, ***p < 0.001, ****p < 0.0001.

SIRT1/2/3 have recently been identified as erasers capable of removing lactyl groups from lysine residues [44]. Given that their downregulation could elevate global protein lactylation, we assessed Sirt1/2/3 expression levels in OGD/R-treated BV2 microglia and observed remarkable decreases in all three sirtuins (Fig. 6A), suggesting impaired lactylation removal in this context. To determine which sirtuin interacts directly with PTBP1, we performed co-immunoprecipitation, which revealed a specific interaction only between SIRT1 and PTBP1 (Fig. 6B). This interaction was substantially weakened in OGD/R-exposed BV2 and HMC3 microglia (Fig. 6C; Supplemental Fig. 6), correlating with elevated PTBP1 lactylation and implying that SIRT1 downregulation compromised PTBP1 delactylation. To functionally validate SIRT1’s role as a delactylase, we silenced SIRT1 in OGD/R-exposed BV2 microglia and observed a marked increase in global lactylation, particularly within the 55–70 kDa range (Fig. 6D). Furthermore, SIRT1 knockdown significantly enhanced PTBP1 lactylation (Fig. 6E), confirming SIRT1 as a direct delactylase for PTBP1. Concomitantly, protein levels of both USP18 and FTO were significantly decreased in SIRT1 knockdown BV2 cells (Fig. 6F), demonstrating that SIRT1 loss exacerbated lactylation-mediated suppression of these downstream effectors. These findings support a feedforward amplification loop in which OGD/R-induced SIRT1 downregulation impairs PTBP1 delactylation, leading to sustained lactylation that suppresses USP18 and FTO expression and further reduces SIRT1 levels, thereby potentiating microglial apoptosis.

**Fig. 7:**
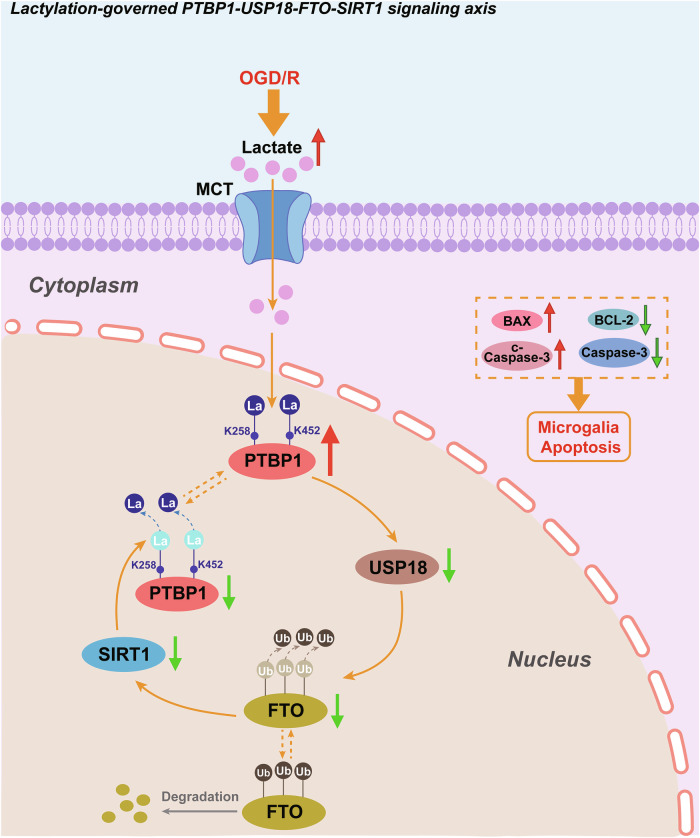
Graphical model of the lactylation-dependent PTBP1-USP18-FTO-SIRT1 signaling axis in regulating microglial apoptosis.

Taken together, our study demonstrates that SIRT1 directly regulates PTBP1 lactylation levels and drives microglial apoptotic signaling through a lactylation-governed PTBP1-USP18-FTO-SIRT1 axis (Fig. 7).

## Discussion

Microglia, the resident immune sentinels of the CNS, serve as critical regulators of neurodevelopment, neural circuit homeostasis, and injury response [12, 13]. Their functional plasticity is tightly modulated by the CNS microenvironment, enabling constant surveillance through phagocytic clearance of pathogens, cellular debris, supernumerary synapses, and toxic protein aggregates that threaten neural integrity [13, 45]. As predominant producers of pro-inflammatory cytokines, microglia constitute pivotal mediators of neuroinflammation, and their dysregulation is implicated in neurodevelopmental disorders, aging-related cognitive decline, and neurodegenerative diseases [45, 46].

Microglial dysfunction exacerbates neural damage post-injury, while apoptosis has emerged as a critical mechanism underlying the pathogenesis and progression of diverse CNS disorders [47, 48]. During early apoptosis, mitochondrial membrane potential collapse and electron transport chain (ETC) impairment diminish oxidative phosphorylation (OXPHOS) efficiency [49], triggering a bioenergetic crisis that shifts cellular metabolism toward accelerated glycolysis and lactate overproduction. Upregulation of key glycolytic enzymes, such as pyruvate kinase M2 (PKM2) and lactate dehydrogenase A (LDHA), along with subsequent lactate accumulation, further promotes apoptotic signaling [50, 51], illustrating a bidirectional link between apoptosis and lactate metabolism that warrants deeper mechanistic investigation. Beyond its metabolic roles, lactate also serves as a substrate for protein lactylation [30], a post-translational modification orchestrating cellular functions and biological processes via histone and non-histone targets [31, 32], and has been implicated in promoting neuronal apoptosis [52].

Cerebral hypoxia and ischemia trigger widespread microglial injuries, with microglial apoptosis representing a critical driver of post-stroke neuroinflammation. Damage-associated molecular patterns (DAMPs) released during acute necrosis activate microglial apoptosis via cGAS-STING signaling [53], exacerbating secondary brain injury. CNS-specific cGAS-knockdown murine stroke models exhibit significantly attenuated DAMP-induced microglial apoptosis with concomitant mitigation of cerebral damage [53], underscoring the pathogenic significance of microglial apoptosis in hypoxic-ischemic pathology. However, the mechanisms regulating microglial apoptosis under these conditions remain incompletely understood.

Here, we investigated the role of lactylation in driving apoptotic signaling in microglia under hypoxic-ischemic conditions. Global lactylome analysis in microglia exposed to OGD/R revealed extensive protein hyperlactylation. Intriguingly, we identified the RNA-binding splicing regulator PTBP1 as a key non-histone target, lactylated at lysine residues K258 and K452 in a manner dynamically regulated by the delactylase SIRT1 and functionally linked to microglial apoptosis.

PTBP1, known for its central roles in post-transcriptional regulation, including alternative splicing, mRNA metabolism, processing, and transport [33, 54], influences diverse biological processes, such as cell differentiation, metabolism, immune response, and apoptosis in a context-dependent manner [54]. It can suppress apoptosis by maintaining AKT activity through PHLDA3 suppression in intestinal epithelial cells [55], or promote apoptosis in gastric cancer cells by stabilizing metabolic enzyme transcripts (such as PGK1) to induce lactate accumulation and oxidative stress [56].

In this study, we demonstrated that hyperlactylated PTBP1 suppressed USP18 expression, leading to FTO degradation and subsequent reduction in SIRT1 levels. This loss of SIRT1-mediated delactylation reinforced PTBP1 lactylation, establishing a self-amplifying loop that drives persistent apoptotic activation. Supporting this model, lactylation-deficient PTBP1 mutants (K258R/K452R) markedly attenuated apoptotic signaling, confirming the functional importance of these modifications. Our findings unveil a lactylation-dependent signaling cascade centered on PTBP1 that critically mediates OGD/R-induced microglial apoptosis, and propose therapeutic intervention targeting the lactylation-governed PTBP1-USP18-FTO-SIRT1 axis as a viable strategy to mitigate apoptosis-related neuropathology.

Emerging evidence from glioma stem cell models positions PTBP1 as an important node in lactylation-mediated glycolytic metabolic regulation, where lactylation of PTBP1-K436 modulates PFKFB4 mRNA stability and metabolic reprogramming [57]. Our findings reveal that upregulation of SIRT1 in response to PTBP1 mutation may represent an adaptive feedback response aimed at restoring lactylation balance. This indicates that the lactylation status of PTBP1 directly influences the cellular delactylation capacity. Together with recent reports demonstrating that lactylation plays multifaceted roles in hypoxia-induced brain damage and neuroinflammation [57–59], our findings identify PTBP1 lactylation as a critical regulator that controls global lactylation dynamics and microglial fate decisions during ischemic injury.

PTBP1 is known to undergo lysine acetylation and lactylation at multiple residues, including sites within its RNA recognition motifs (RRMs), which can modulate its RNA-binding properties and splicing activity [60, 61]. Notably, we identified the lysine residues as the primary lactylation site (K258 and K452). Given that both acetylation and lactylation occur on lysine residues, these modifications may compete for the same site or exert opposing effects on PTBP1 conformation and function. It is plausible that the functional outcome, enhanced RNA-binding capacity and pro-apoptotic signaling, reflects the relative abundance and dynamics of these two modifications under OGD/R conditions. The predominance of lactylation in our system may be driven by the substantial lactate accumulation following oxygen-glucose deprivation, which shifts the equilibrium toward lactylation. This metabolic context may therefore determine which modification predominates and the direction of PTBP1 functional regulation. Future studies aimed at systematically mapping the PTBP1 modification landscape under different stress conditions and dissecting the competitive or cooperative relationships among these modifications will be essential to fully understand how this pleiotropic RNA-binding protein integrates diverse cellular signals to coordinate gene expression programs.

The emerging field of lactylation research has revealed complex, context-dependent roles for this modification in regulating apoptosis. Recent studies demonstrate that lactylation can either promote or inhibit cellur apoptosis depending on cellular context, metabolic status, and specific protein targets [52, 62]. Mechanistically, non-histone lactylation has been shown to directly modulate apoptotic regulators, thereby dictating cell fate outcomes. For instance, caspase-3 lactylation impedes its activation and suppresses apoptosis [63], while lactylation of p53 at K120 disrupts its pro-apoptotic function [64]. Our finding that PTBP1 lactylation promotes microglial apoptosis under OGD/R conditions adds an important dimension to this framework. While this pro-apoptotic role contrasts with several reports describing lactylation as an apoptosis-suppressive modification in cancer contexts, we propose that this discrepancy reflects fundamental differences in cell type, metabolic microenvironment, and substrate specificity. These considerations position our findings within a broader conceptual model in which lactylation serves as a dynamic metabolic sensor that translates microenvironmental lactate flux into context-specific cell-fate decisions.

Translationally, our findings suggest a paradigm shift from targeting individual molecules to modulating the lactylation process itself, revealing that functional coordination of PTBP1, USP18, FTO, and SIRT1 is governed by PTBP1 lactylation, a metabolic stress sensor integrating microenvironmental lactate signals into coordinated cellular responses. This insight opens innovative therapeutic avenues: developing inhibitors against the responsible lactyltransferase to block PTBP1 lactylation at its origin, or activating the “eraser” SIRT1 with compounds, such as SRT1720 [65], which has shown efficacy in cerebral ischemia by reducing protein lactylation. Compared to direct PTBP1 knockdown, which may perturb its diverse physiological functions in RNA metabolism, targeting its lactylation offers superior reversibility and spatiotemporal controllability, preserving normal PTBP1 function while blocking pathological activation under ischemic stress. Beyond therapeutic intervention, the context-dependent nature of lactylation supports the development of a multi-marker molecular stratification system that combines PTBP1 lactylation status, USP18 levels, FTO expression, and SIRT1 activity to generate a predictive signature for patient outcomes and therapeutic responsiveness, thereby advancing precision medicine in ischemic stroke.

Taken together, our study addresses an important mechanistic gap in hypoxic-ischemic neuropathology by elucidating the lactylation-driven post-translational regulation of PTBP1 and delineating its downstream interaction networks within microglia under hypoxic-ischemic stress, offering both novel mechanistic insights into neuroinflammatory apoptosis and promising therapeutic targets for hypoxic-ischemic neurological disorders.

## Conclusion

In conclusion, this study reveals that the OGD/R induces pronounced lactylation of PTBP1 in microglia. We demonstrate that hyperlactylated PTBP1 suppresses key downstream regulators, including USP18, FTO, and SIRT1, thereby activating intrinsic apoptotic pathways. Additionally, we identify a positive feedback loop in which SIRT1 deficiency enhances PTBP1 lactylation, further amplifying microglial apoptosis. These findings elucidate a novel lactylation-dependent circuit that drives hypoxic-ischemic injury and provide the lactylation-governed PTBP1-USP18-FTO-SIRT1 signaling axis as a promising therapeutic target for mitigating microglia-mediated neuropathology in cerebral ischemia and related conditions.

## Supplementary information

Supplemental File 1

Supplemental Material

Supplemental Figure 1

Supplemental Figure 2

Supplemental Figure 3

Supplemental Figure 4

Supplemental Figure 5

Supplemental Figure 6

Original Data

## Data Availability

Data supporting the findings of this study are available from the corresponding authors upon reasonable request.
