# Supplementary material for: Lactylation of PTBP1 drives a pro-apoptotic positive feedback loop in microglia following oxygen-glucose deprivation/reoxygenation-induced injury
Source: Cell Death Dis. 2026 May 28;17(1):658. doi: 10.1038/s41419-026-08921-9 (PMC13402602; doi:10.1038/s41419-026-08921-9)
Supplement: Supplementary file 1 — Supplemental File 1 [file 41419_2026_8921_MOESM1_ESM.docx]

**Supplemental File 1****. Full-length protein sequence of PTBP1 (Mus musculus, 555 aa, UniProtKB:P17225).** The lactylated lysine residues K258 and K452 are indicated in red.

MDGIVPDIAVGTKRGSDELFSTCVSNGPFIMSSSASAANGNDSKKFKGDNRSAGVPSRVIHVRKLPSDVTEGEVISLGLPFGKVTNLLMLKGKNQAFIEMNTEEAANTMVNYYTSVAPVLRGQPIYIQFSNHKELKTDSSPNQARAQAALQAVNSVQSGNLALAASAAAVDAGMAMAGQSPVLRIIVENLFYPVTLDVLHQIFSKFGTVLKIITFTKNNQFQALLQYADPVSAQHAKLSLDGQNIYNACCTLRIDFSKLTSLNVKYNNDKSRDYTRPDLPSGDSQPSLDQTMAAAFGAPGIMSASPYAGAGFPPTFAIPQAAGLSVPNVHGALAPLAIPSAAAAAAASRIAIPGLAGAGNSVLLVSNLNPERVTPQSLFILFGVYGDVQRVKILFNKKENALVQMADGSQAQLAMSHLNGHKLHGKSVRITLSKHQSVQLPREGQEDQGLTKDYGSSPLHRFKKPGSKNFQNIFPPSATLHLSNIPPSVSEDDLKSLFSSNGGVVKGFKFFQKDRKMALIQMGSVEEAVQALIELHNHDLGENHHLRVSFSKSTI
