# Supplementary material for: Lactylation of PTBP1 drives a pro-apoptotic positive feedback loop in microglia following oxygen-glucose deprivation/reoxygenation-induced injury
Source: Cell Death Dis. 2026 May 28;17(1):658. doi: 10.1038/s41419-026-08921-9 (PMC13402602; doi:10.1038/s41419-026-08921-9)
Supplement: Supplementary file 2 — Supplemental Material [file 41419_2026_8921_MOESM2_ESM.docx]

**Supplemental Figures**

**Supplemental Figure 1. Hypoxic-ischemic conditions drive protein lactylation and induce apoptosis in HMC3 cells. A** Western blot analysis of global lysine acetylation (Pan-Kla) in HMC3 cells following OGD/R. **B** Ki67 staining in HMC3 cells under control and OGD/R conditions. Scale bars, 100 μm. **C** Western blot analysis of BCL-2, BAX, cleaved Caspase-3, and total Caspase-3 in HMC3 cells. **D** TUNEL staining in HMC3 cells under control and OGD/R conditions. Scale bars, 20 μm. Unpaired two-tailed Student’s t-tests are applied. All quantitative data, derived from at least three independent biological replicates, are presented as mean ± SD. *p < 0.05, **p < 0.01, ***p < 0.001.

**Supplemental Figure 2.** **The number of significantly differentially lactylated proteins and sites identified in BV2 cells under OGD/R conditions compared to the control group.**

**Supplemental Figure 3.** **The MS/MS spectra of the lactylated peptides “IDFS(Kla)LTSLNVK” and “EGQEDQGLT(Kla)DYGSSPLHR”.**

**Supplemental Figure 4. PTBP1 lactylation regulates Usp18 expression through modulation of mRNA-binding stability. A** Relative expression levels of Usp18 mRNA detected by qPCR. **B** RNA immunoprecipitation (RIP) assay followed by qPCR analysis showing the relative binding of PTBP1 to Usp18 mRNA in BV2 cells under normoxia versus OGD/R conditions. Unpaired two-tailed Student’s t-tests are applied. All quantitative data, derived from at least three independent biological replicates, are presented as mean ± SD. *p < 0.05.

**Supplemental Figure 5.** **Diagram of FTO acetylation sites.**

**Supplemental Figure 6. Western blot analysis of PTBP1 lactylation and PTBP1-SIRT1 interaction in HMC3 cells.** Unpaired two-tailed Student’s t-tests are applied. All quantitative data, derived from three independent biological replicates, are presented as mean ± SD. **p < 0.01.
