# Supplementary figures and images for: Lactylation of PTBP1 drives a pro-apoptotic positive feedback loop in microglia following oxygen-glucose deprivation/reoxygenation-induced injury
Source: Cell Death Dis. 2026 May 28;17(1):658. doi: 10.1038/s41419-026-08921-9 (PMC13402602; doi:10.1038/s41419-026-08921-9)

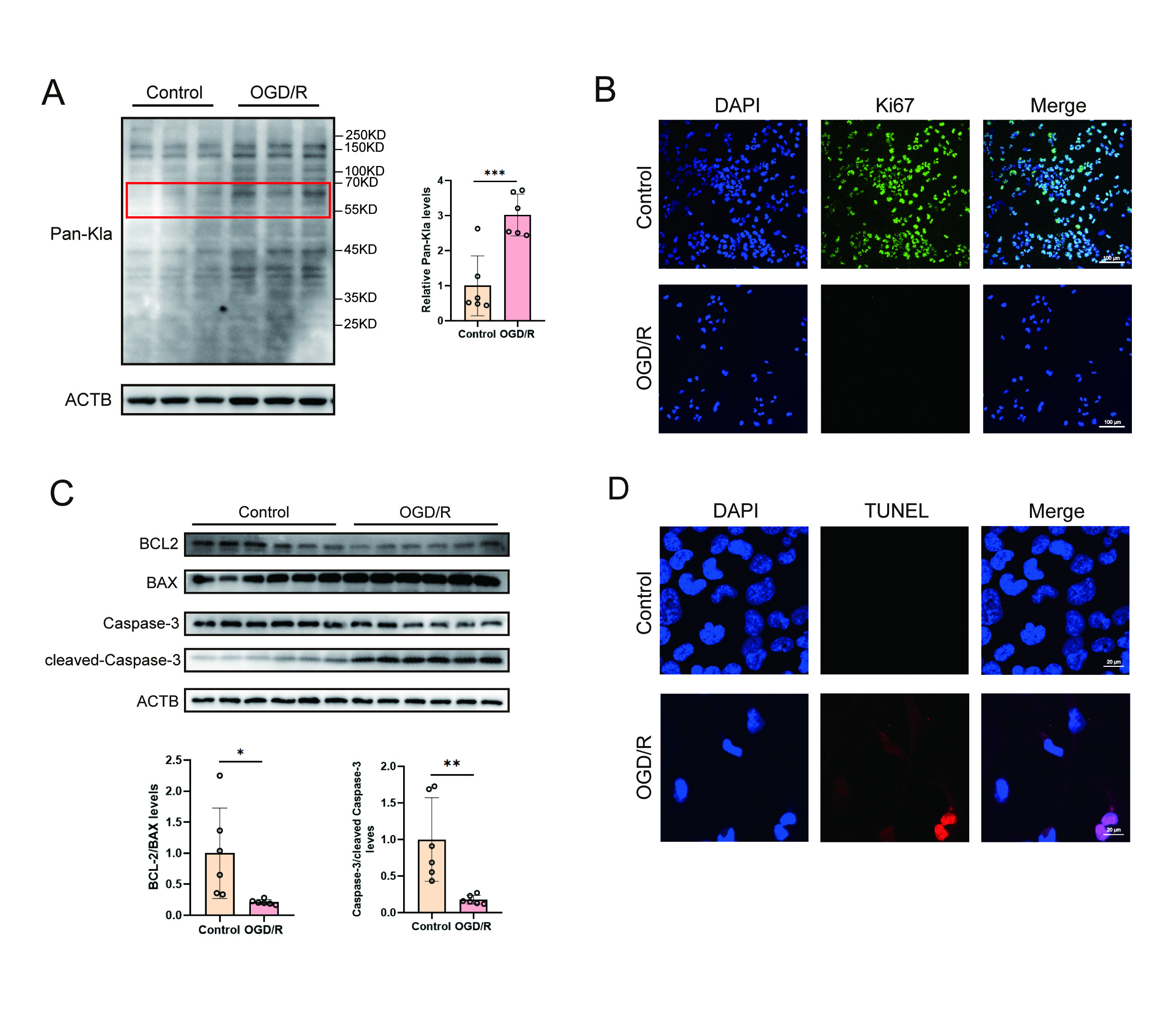

Supplement: Supplementary file 3 — Supplemental Figure 1 [file 41419_2026_8921_MOESM3_ESM.jpg]

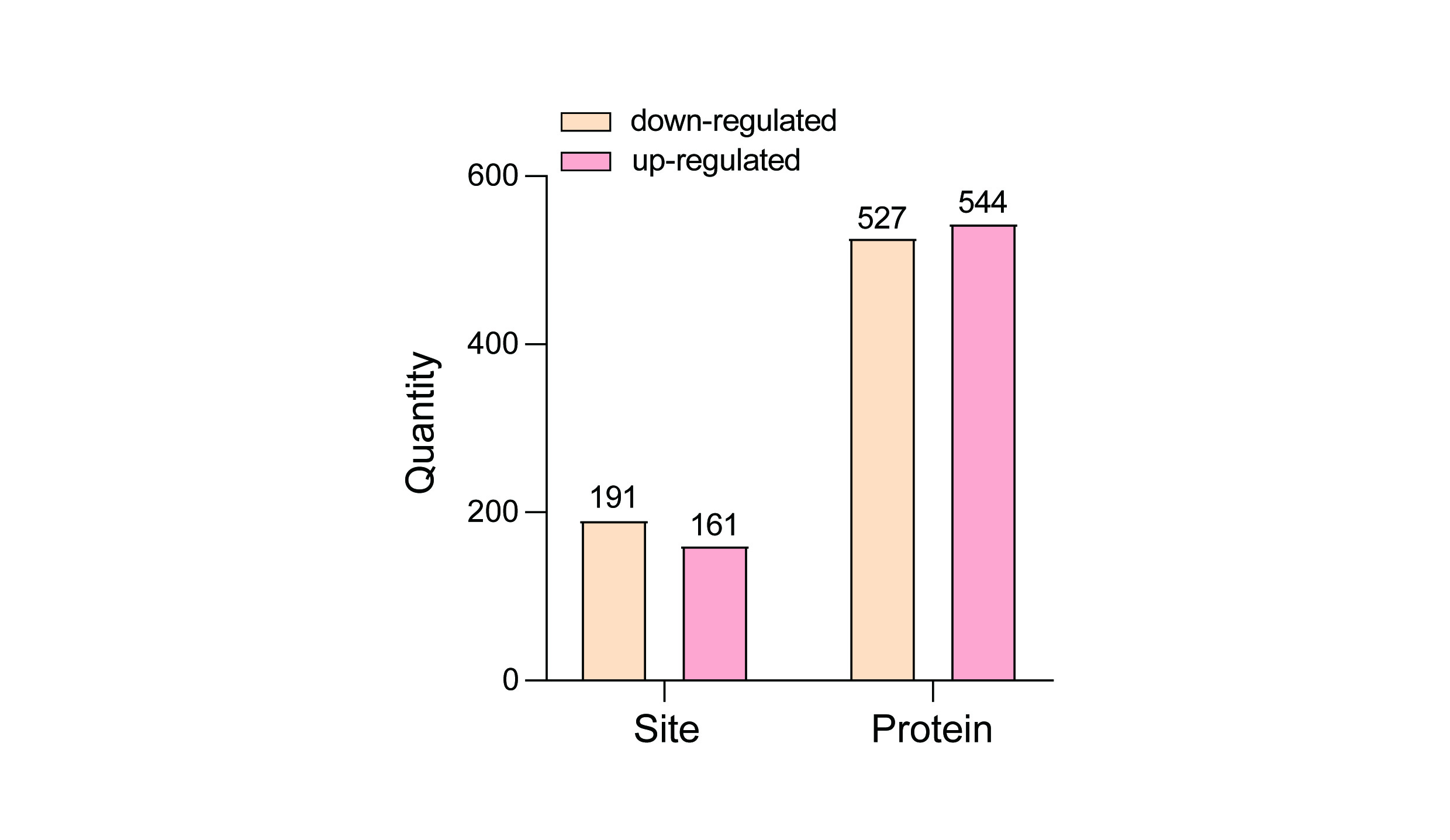

Supplement: Supplementary file 4 — Supplemental Figure 2 [file 41419_2026_8921_MOESM4_ESM.jpg]

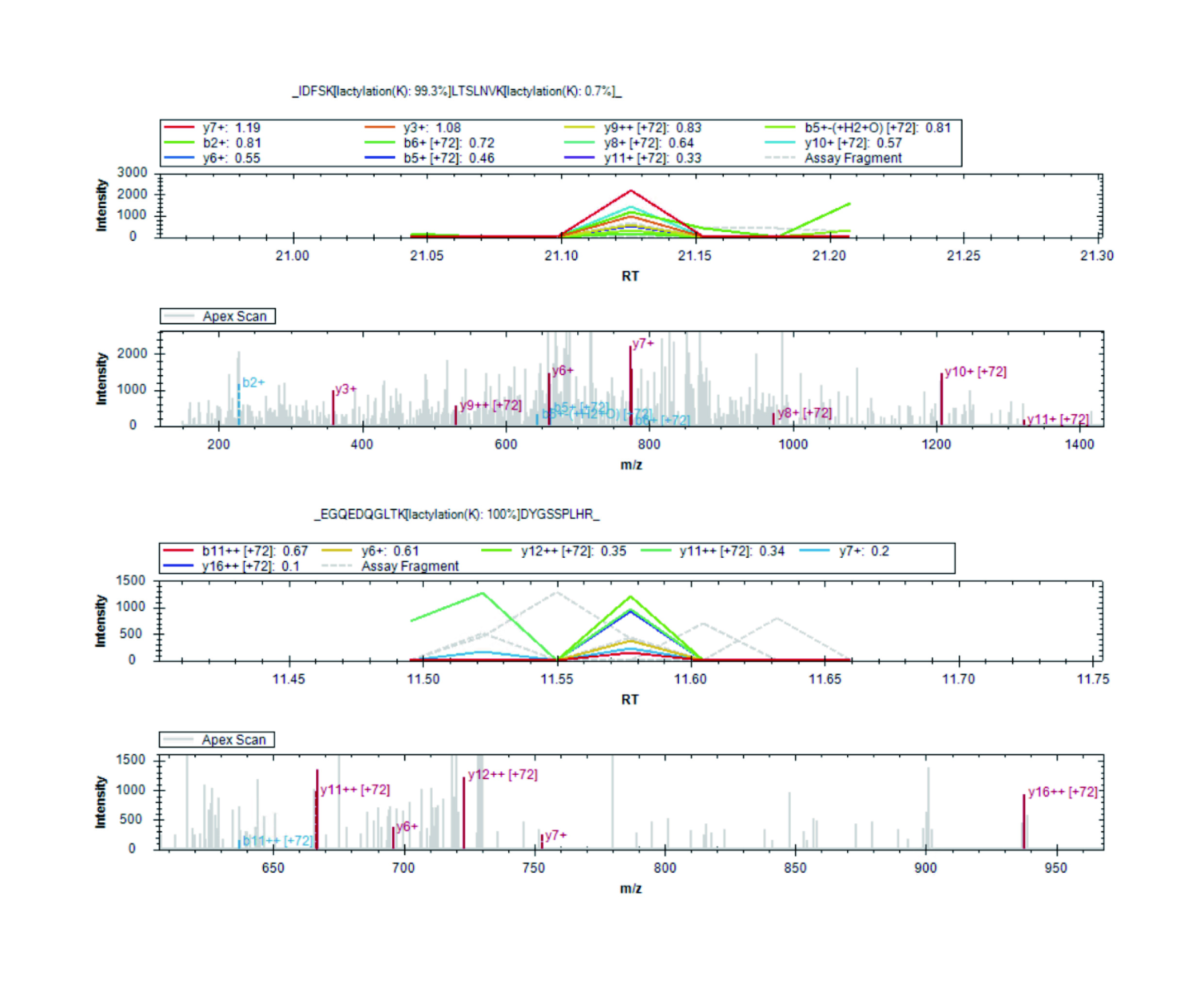

Supplement: Supplementary file 5 — Supplemental Figure 3 [file 41419_2026_8921_MOESM5_ESM.jpg]

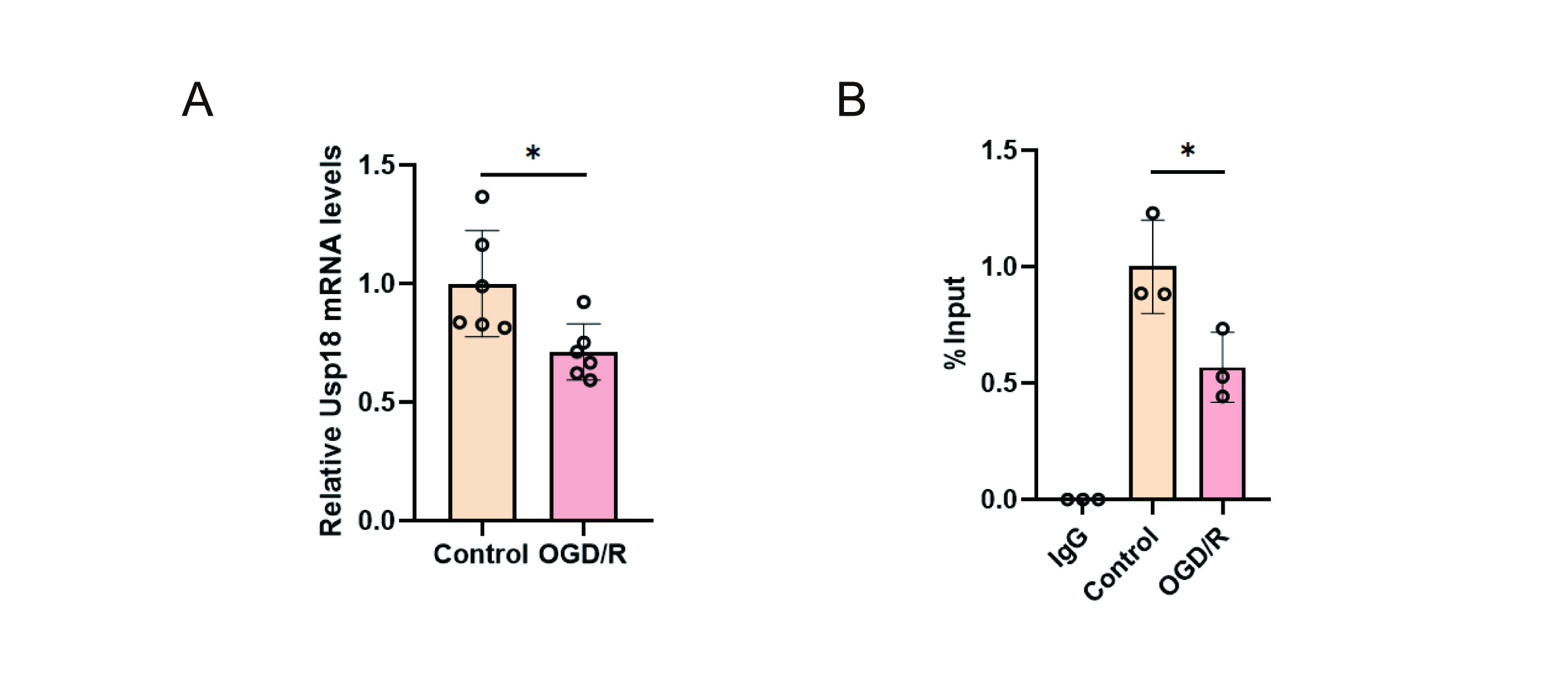

Supplement: Supplementary file 6 — Supplemental Figure 4 [file 41419_2026_8921_MOESM6_ESM.jpg]

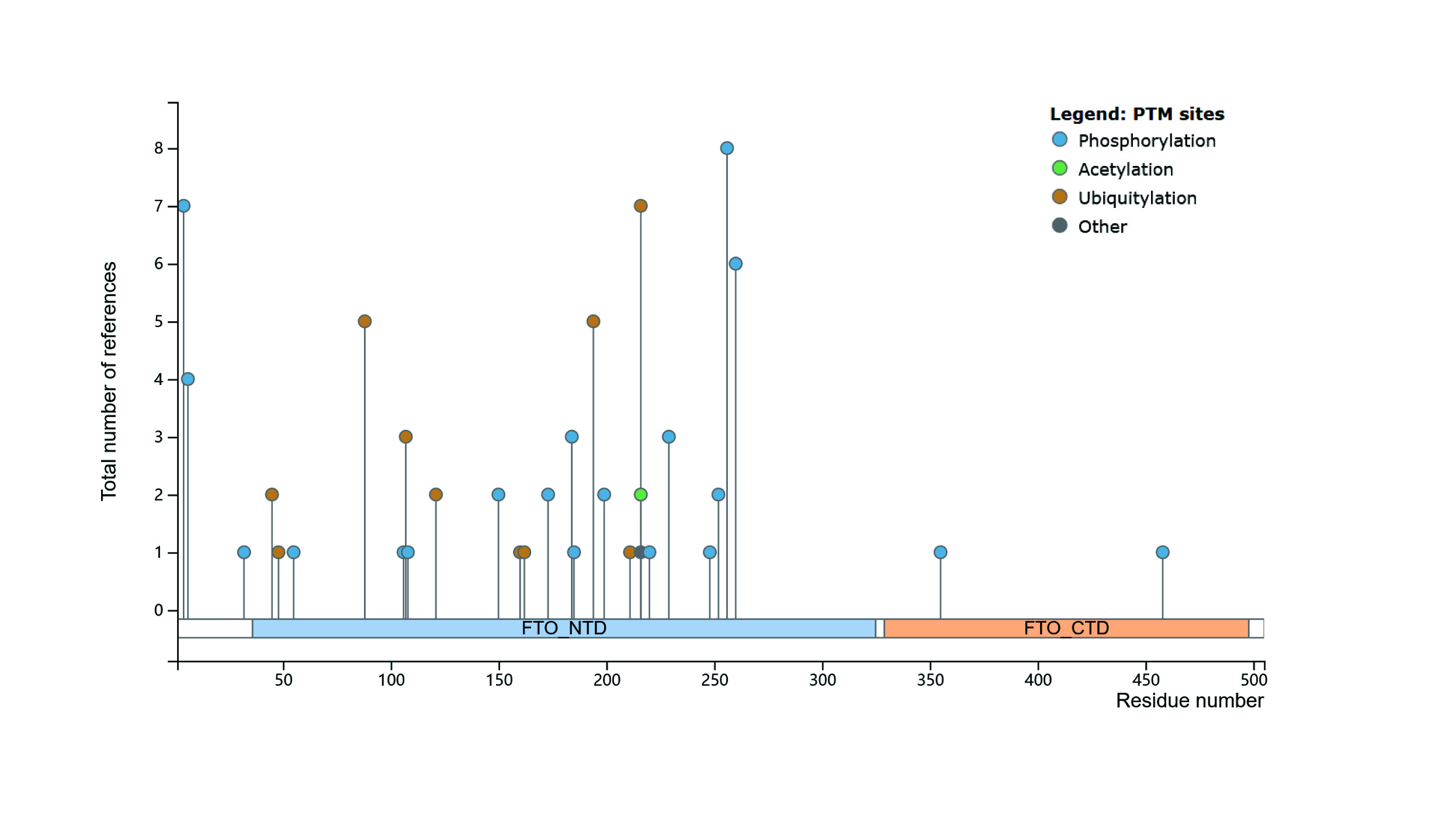

Supplement: Supplementary file 7 — Supplemental Figure 5 [file 41419_2026_8921_MOESM7_ESM.jpg]

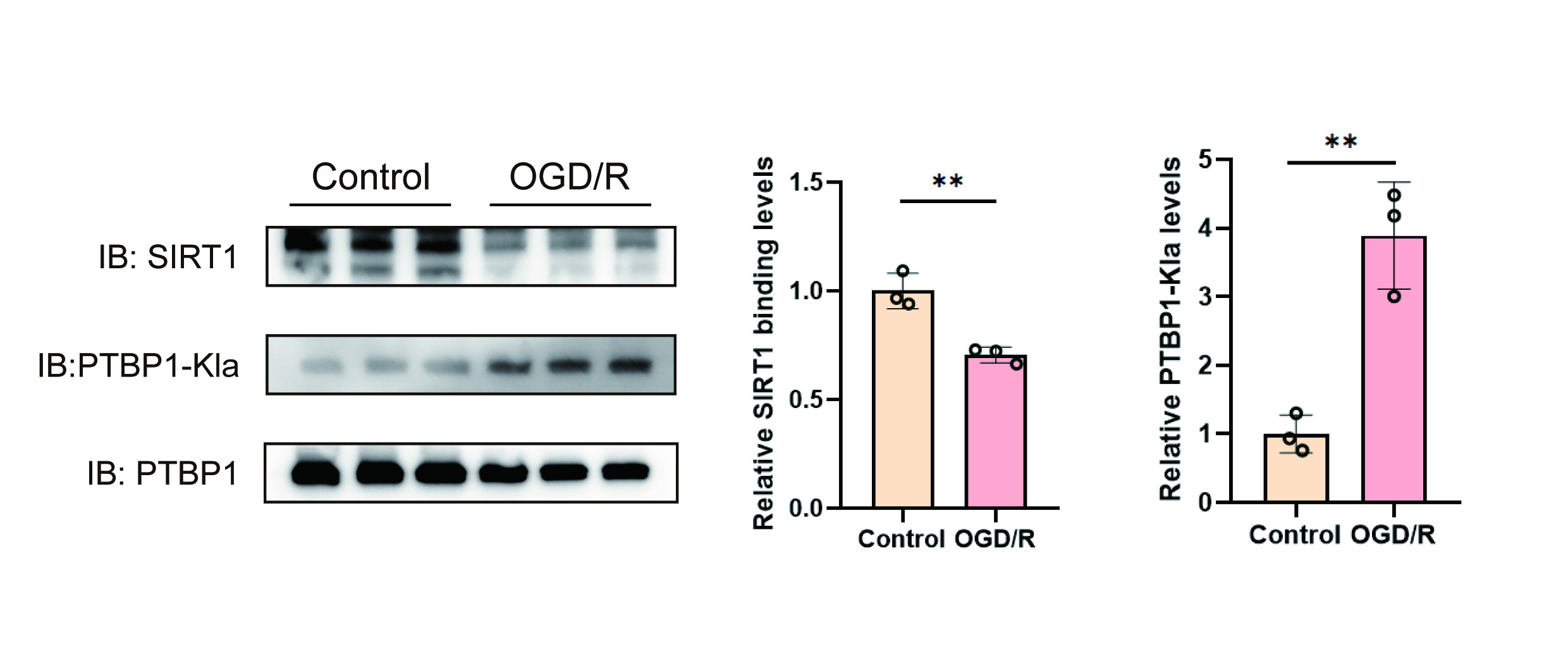

Supplement: Supplementary file 8 — Supplemental Figure 6 [file 41419_2026_8921_MOESM8_ESM.jpg]
