# Supplementary material for: Lactylation of PTBP1 drives a pro-apoptotic positive feedback loop in microglia following oxygen-glucose deprivation/reoxygenation-induced injury
Source: Cell Death Dis. 2026 May 28;17(1):658. doi: 10.1038/s41419-026-08921-9 (PMC13402602; doi:10.1038/s41419-026-08921-9)
Supplement: Supplementary file 9 — Original Data [file 41419_2026_8921_MOESM9_ESM.pdf]

Figure.1

Fig1B

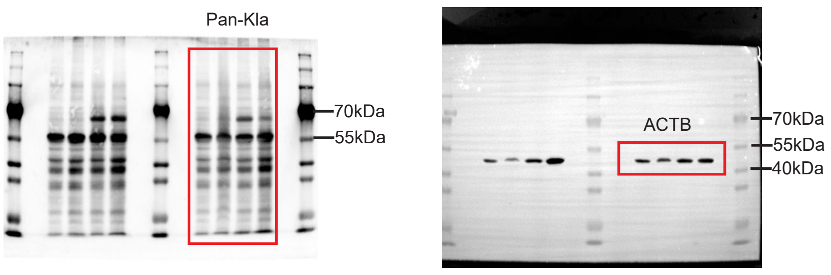

Fig1F

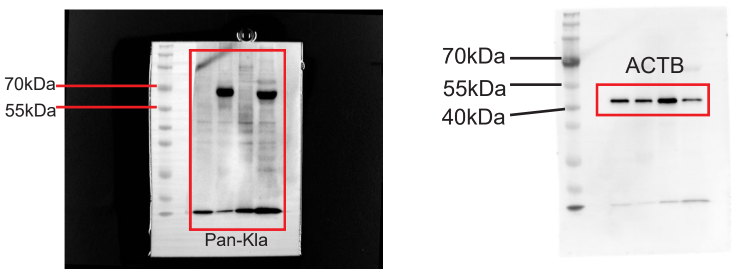

Fig1J

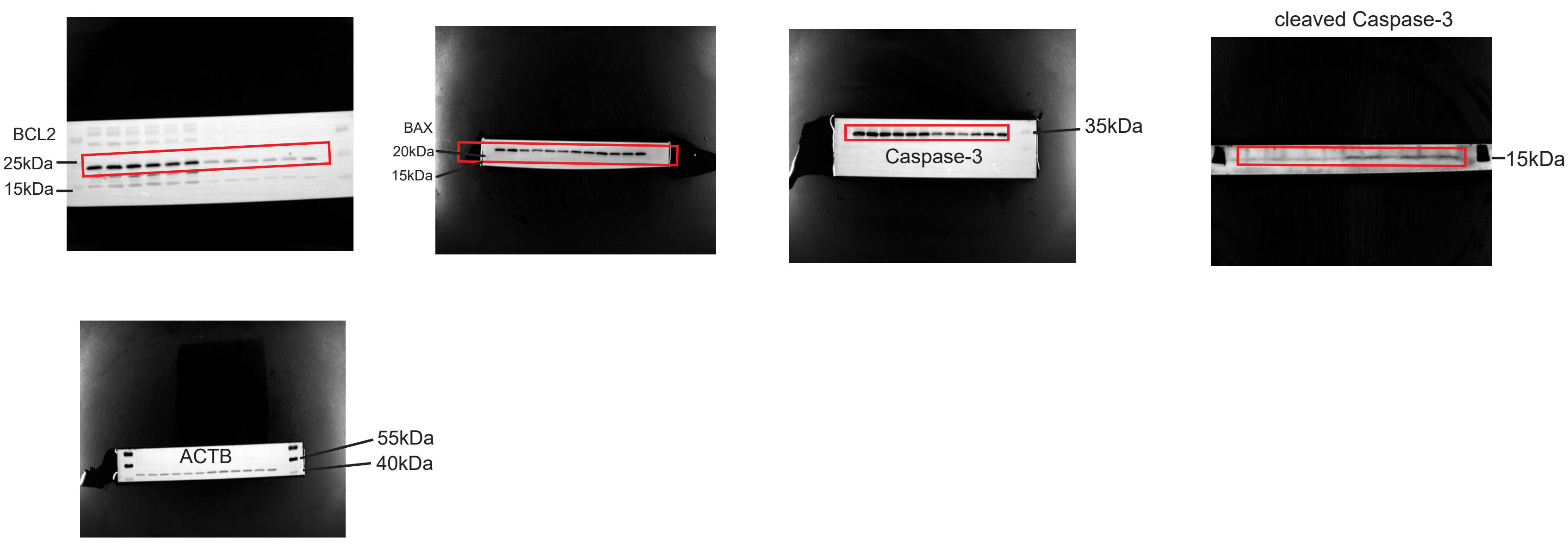

Figure.3

Fig3A

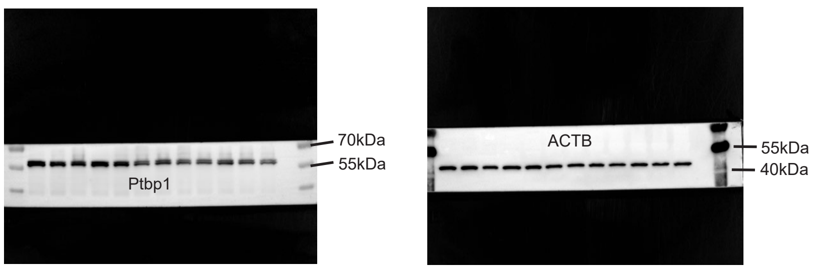

Fig3B

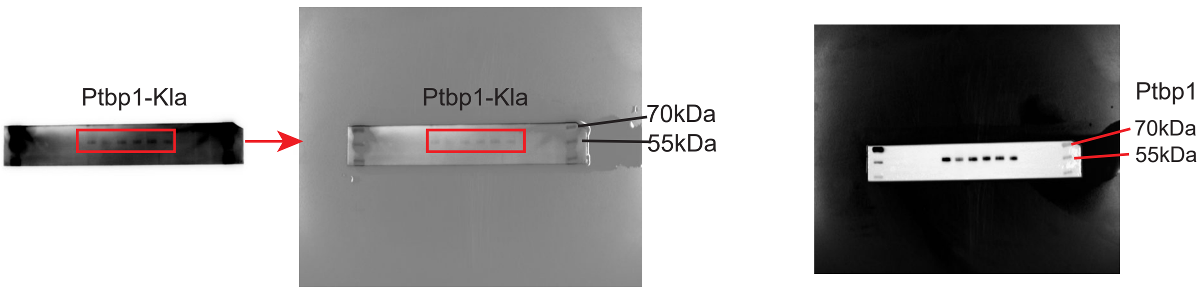

Fig3C

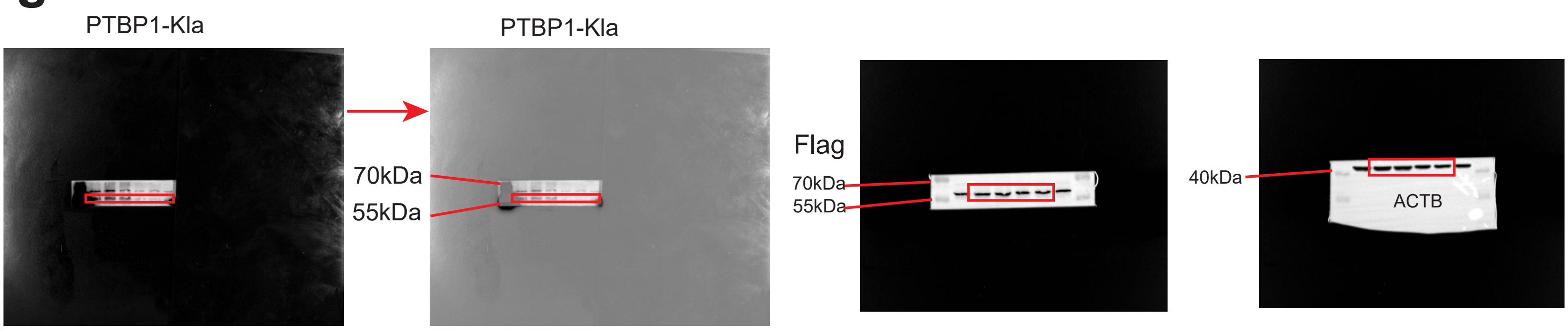

Fig3F

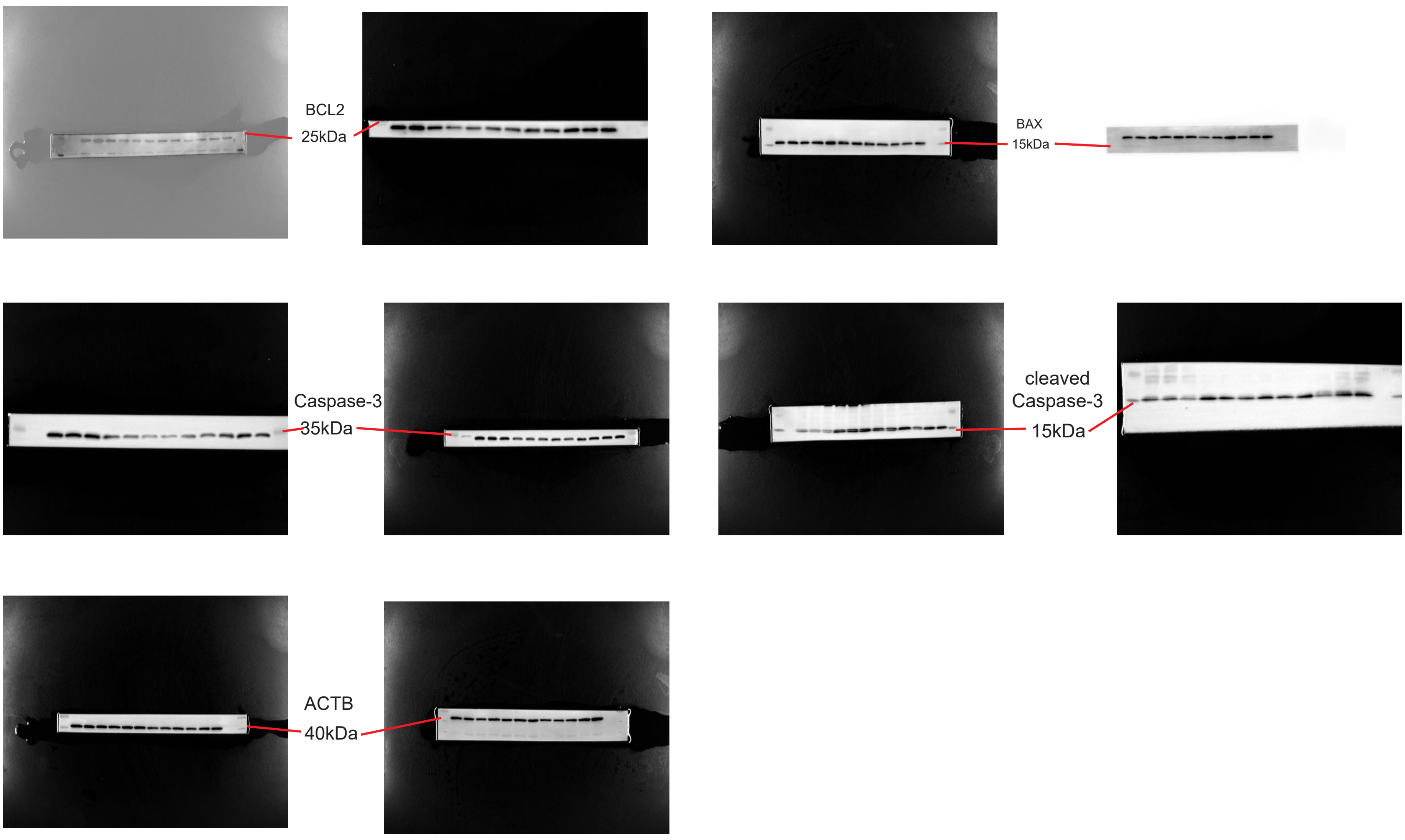

Figure.4

Fig4H

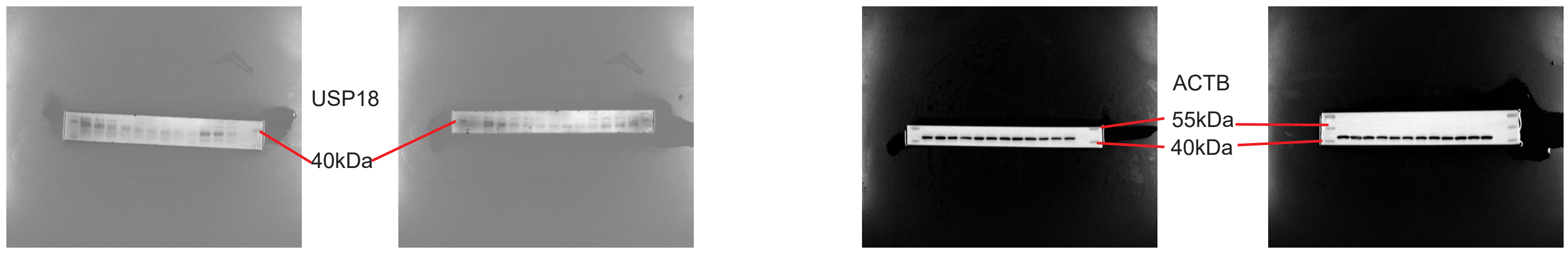

Figure.5

Fig5A

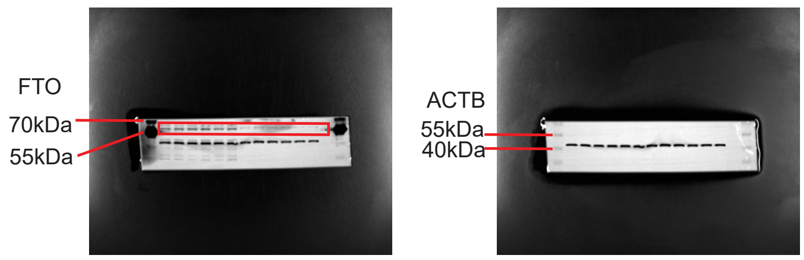

Fig5B

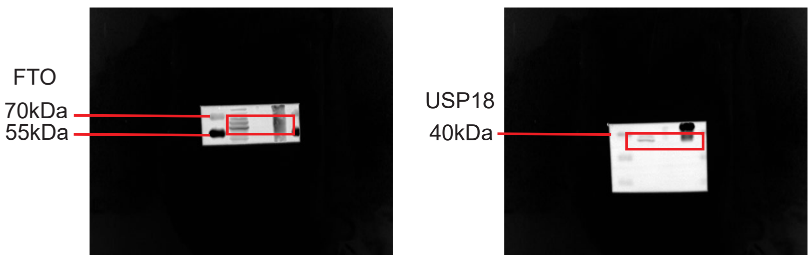

Fig5C

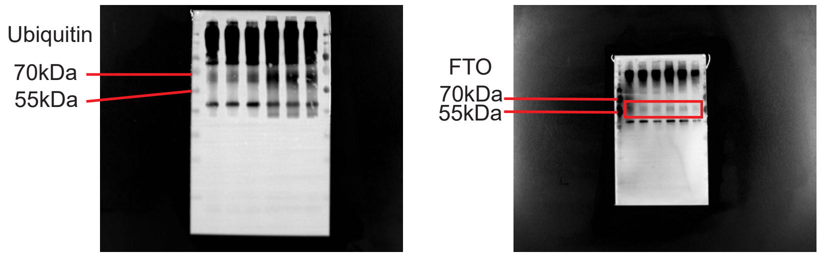

Fig5D

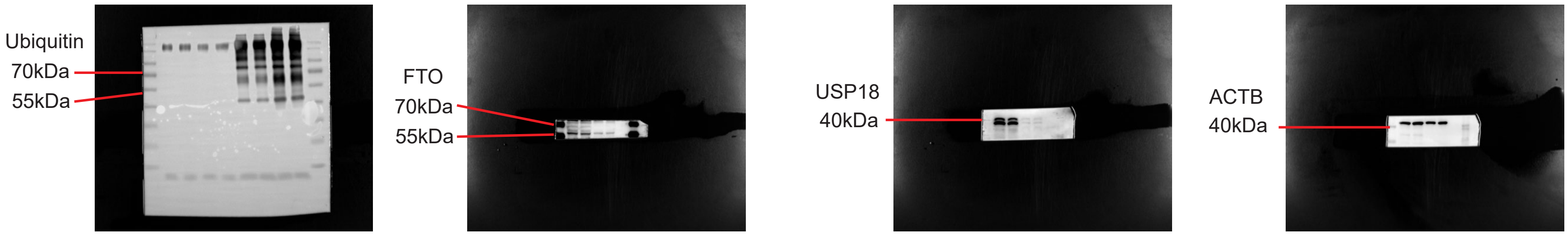

Fig5E

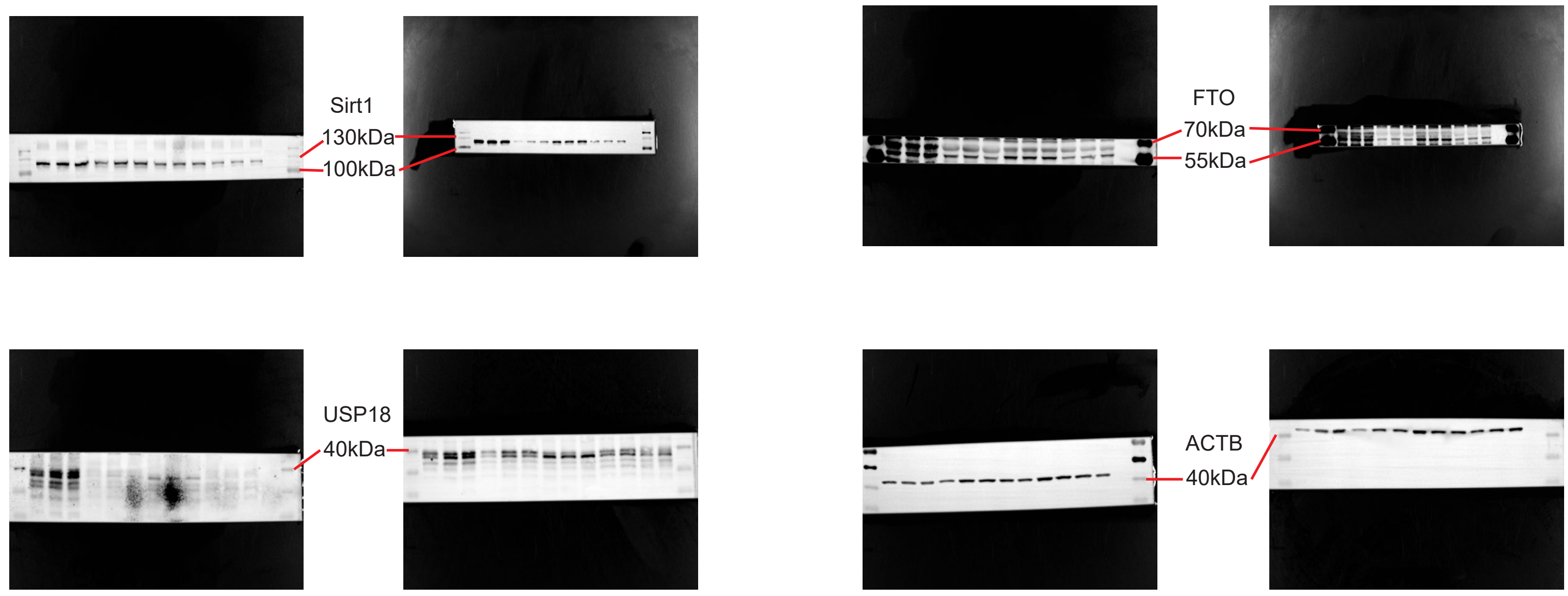

Fig5F

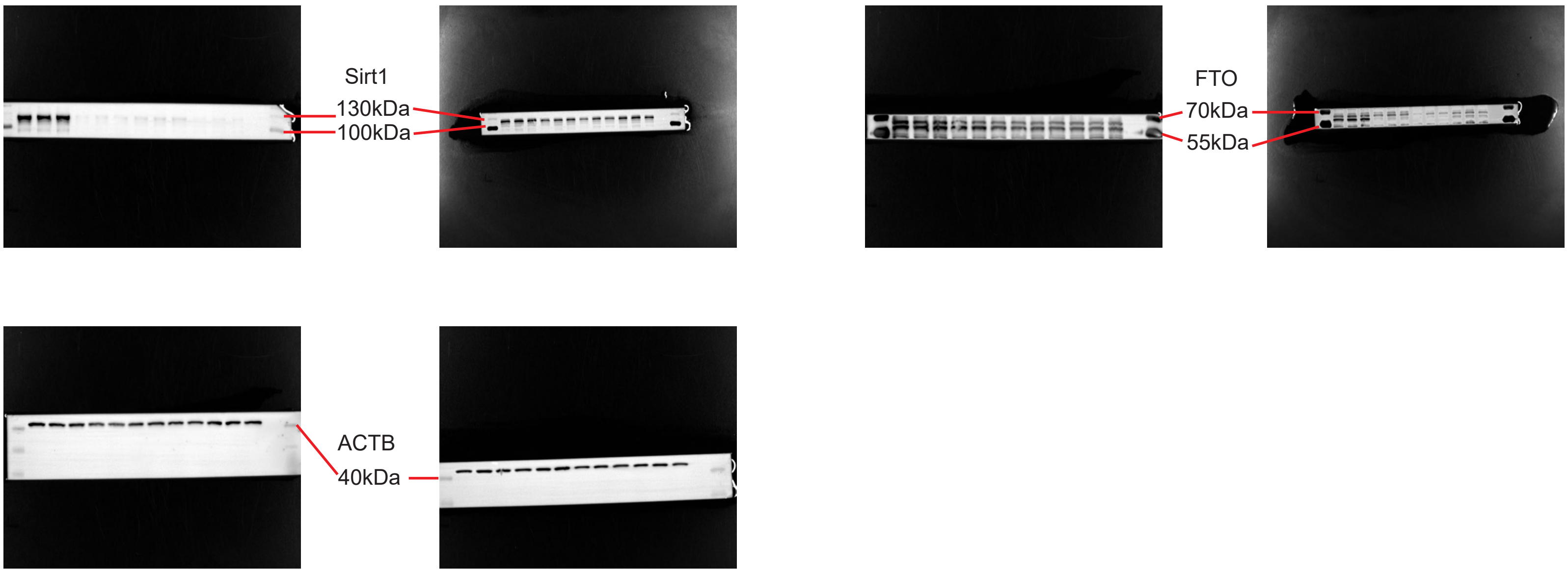

Fig5G

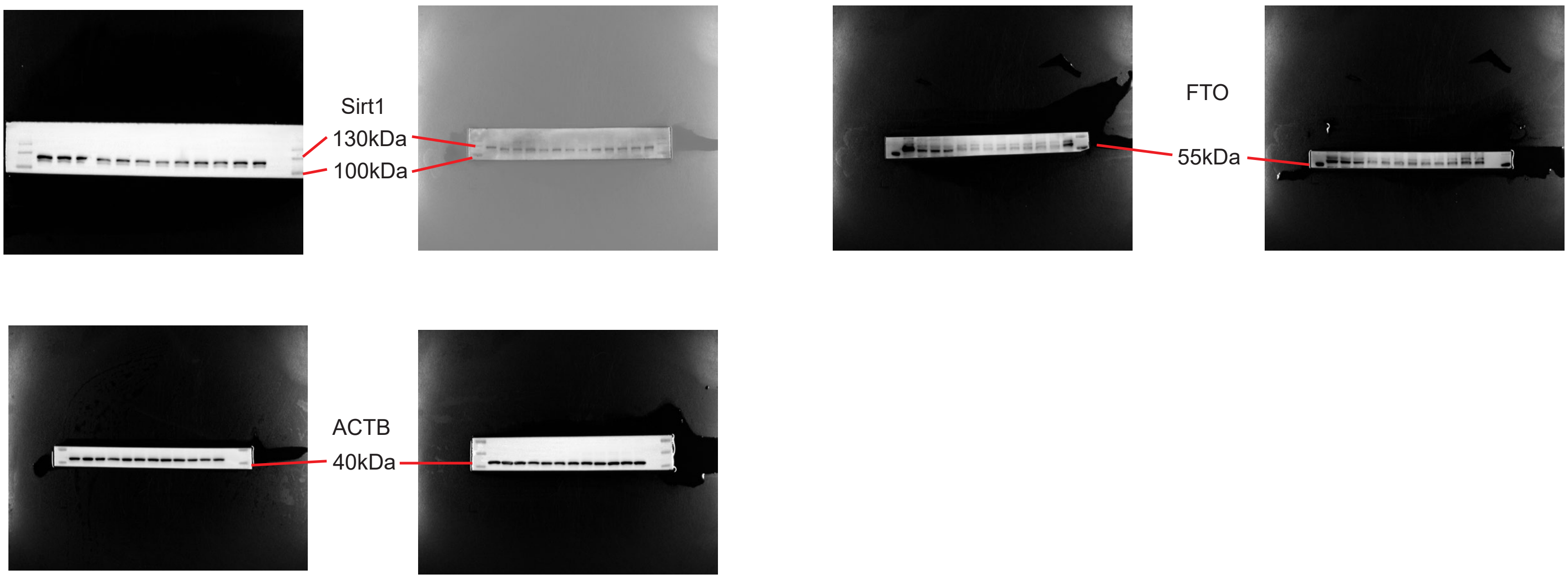

Fig5I

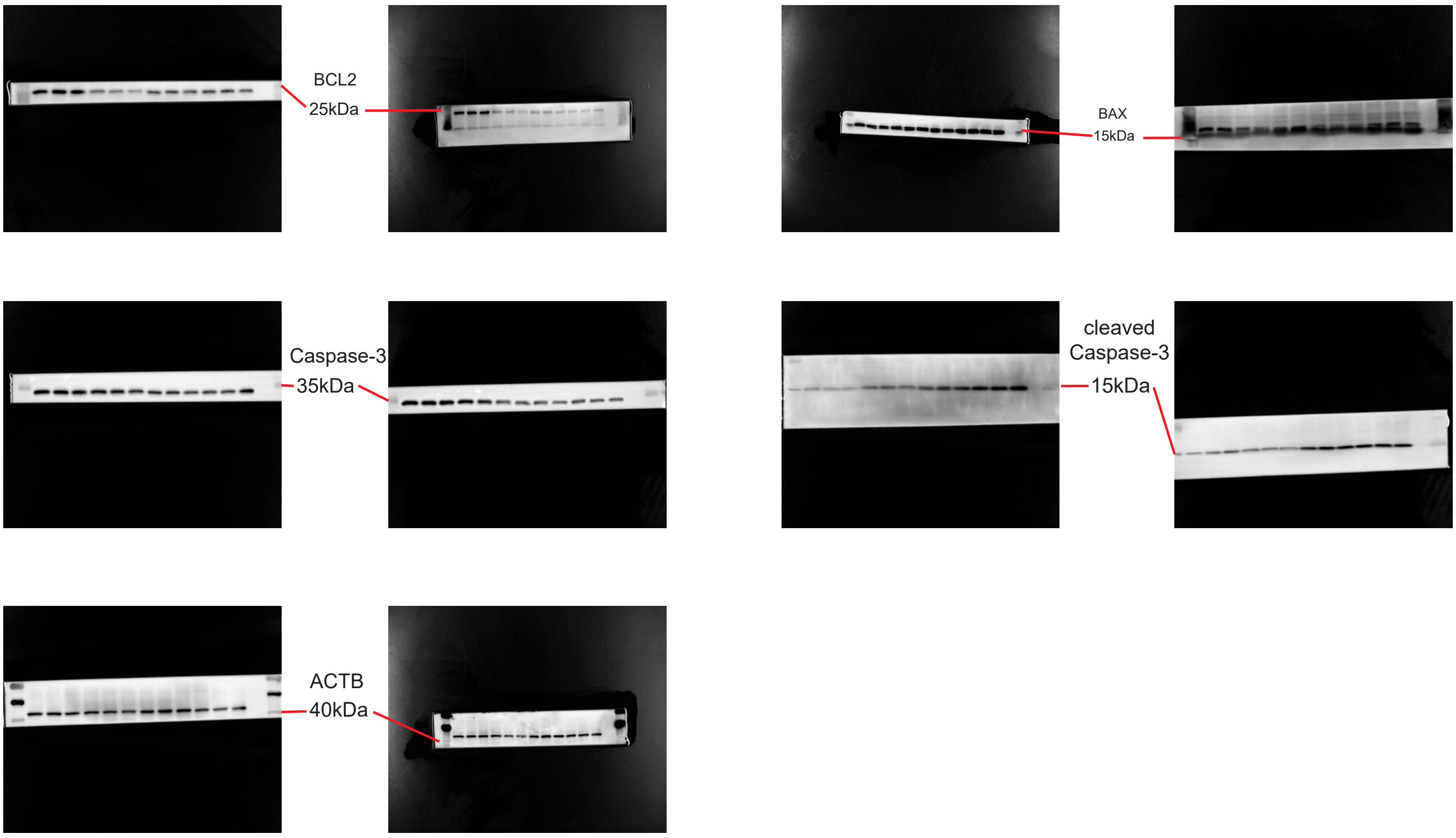

Figure.6

Fig6A

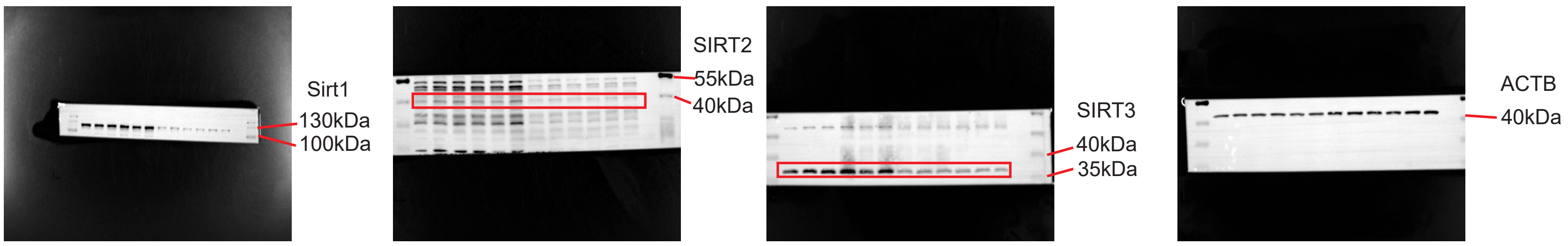

Fig6B

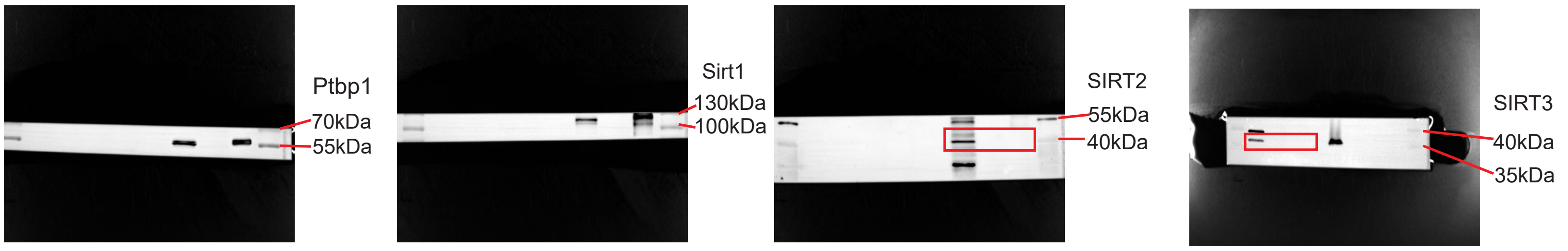

Fig6C

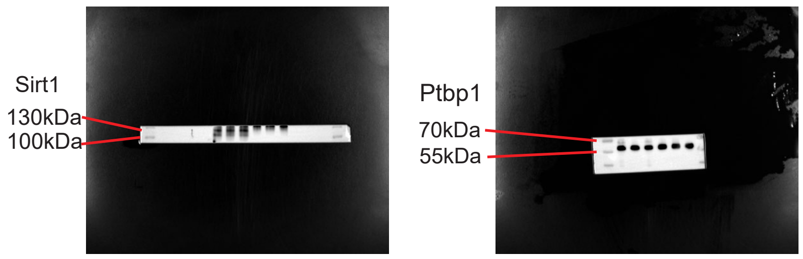

Fig6D

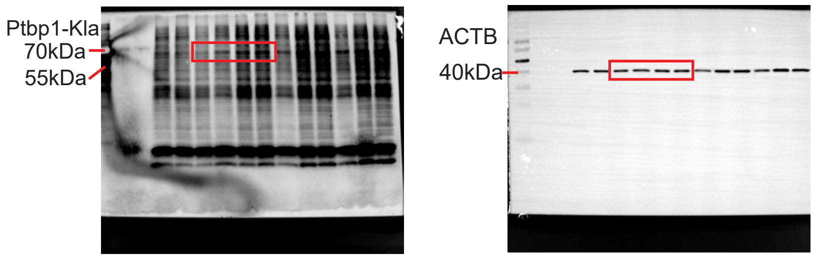

Fig6E

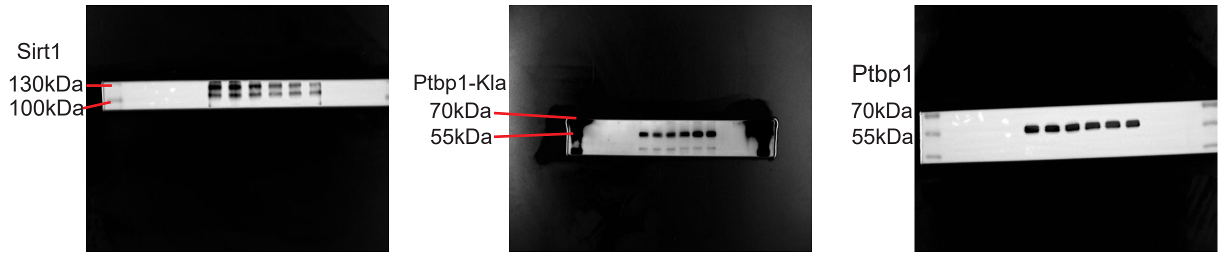

Fig6F

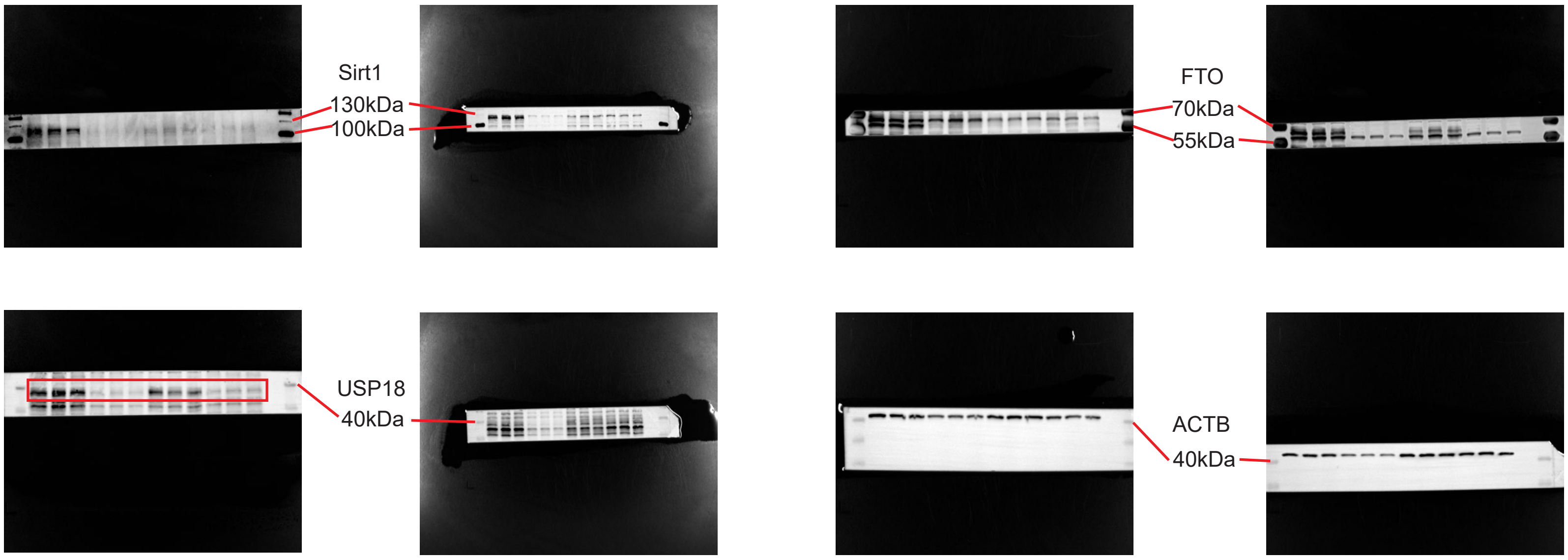

Supplemental Fig.1A

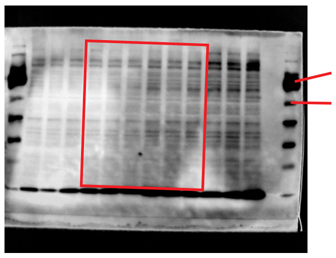

Kla
70kDa
55kDa

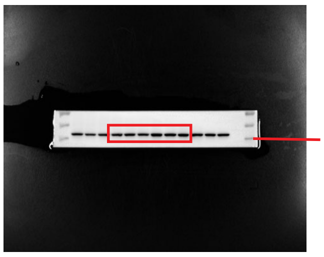

ACTB
40kDa

Supplemental Fig.1C

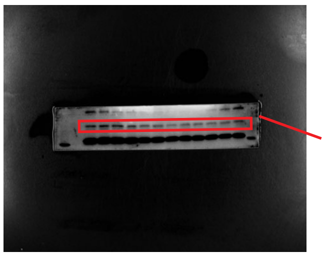

BCL2
25kDa

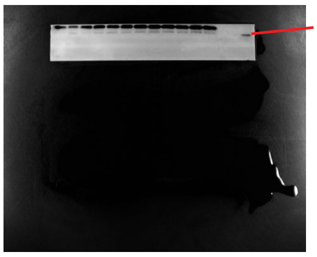

BAX
15kDa

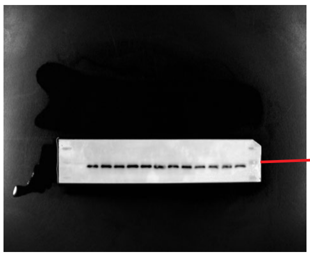

Caspase-3
35kDa

cleaved
Caspase-3
15kDa

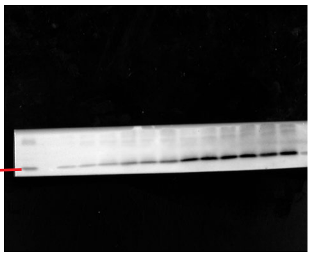

ACTB
40kDa

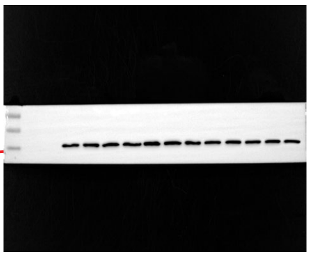

Supplemental Fig.6

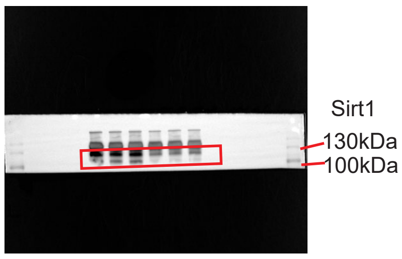

Sirt1
130kDa
100kDa

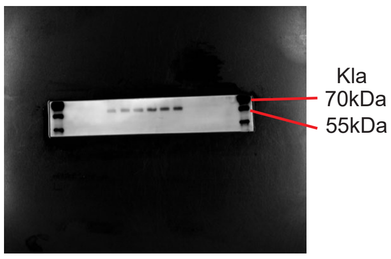

Kla
70kDa
55kDa

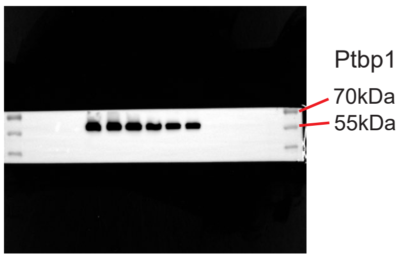

Ptbp1
70kDa
55kDa
